# Reselling or agency selling? Sales mode selection for a manufacturer in private label competition under information asymmetry

**DOI:** 10.1371/journal.pone.0341467

**Published:** 2026-02-03

**Authors:** Min Yang, Anqi Li, Yang Yang

**Affiliations:** 1 College of Economics and Management, China Three Gorges University, Yichang, China; 2 Business School, University of St Andrews, St Andrews, United Kingdom; 3 School of Economics and Management, Yunnan Minzu University, Kunming, China; Czech University of Life Sciences Prague: Ceska Zemedelska Univerzita v Praze, CZECHIA

## Abstract

Big data systems enhance retailers’ predictive capabilities, providing them with a significant advantage in understanding end-market demand, increasing the competitiveness of retailers’ private labels, and significantly impacting manufacturers’ sales strategies. This paper applies dynamic game-theoretic models to explore the interaction between the retailer’s information-sharing strategies and the manufacturer’s sales mode selections when the retailer introduces a private label. Through backward induction, the equilibrium strategies of both parties can be derived. The results show that the manufacturer consistently benefits from information-sharing under both sales modes, potentially achieving a win-win outcome under the agency selling mode. Improving the retailer’s information forecast accuracy encourages a shift from reselling to agency selling mode. The retailer’s lower information forecast accuracy or the smaller demand variance offers no first-mover advantage to either the manufacturer or the retailer. Additionally, an Information Compensation Mechanism (ICM) for revenue-sharing is proposed, and contract conditions across different sales modes are discussed.

## 1. Introduction

Retail commerce has grown significantly, with global sales reaching $26.4 trillion in 2021 and expected to hit $31.1 trillion by 2024 [1]. The agency selling mode is commonly employed in routine retail operations, alongside the traditional reselling mode. In the retailing mode, platforms acquire merchandise from manufacturers and subsequently provide these products to end-market consumers. For instance, JD.com, a globally recognized retailer, redistributes acquired goods to consumers via its self-run stores, accounting for over 82.7% of its total revenue in 2022 [2]. In the agency selling mode, manufacturers engage in direct sales to consumers via retailers and compensate retailers with a consignment fee per transaction. For instance, Taobao, one of the leading e-commerce platforms in the Asia-Pacific region, charges a service fee to third-party sellers, equivalent to 0.6% of the transaction value conducted on its platform [3].

Many large retailers, besides selling manufacturers’ products, introduce private labels that directly compete with these offerings to enhance profitability [4,5]. By the end of 2022, Freshippo, a well-known retailer in China, had its private label products account for 35% of total sales, with ten Freshippo private labels generating over 100 million yuan (around 14 million USD) in revenue [6]. Simultaneously, compared with manufacturers, retailers are closer to consumers and can capitalize on multi-source consumer data. By employing advanced techniques such as machine learning [7], deep learning [8], and hybrid methods [9], retailers are able to build big data platforms, generate demand forecasts [10], and thereby secure competitive advantages for their private labels. For example, JD.com utilizes its big data platform to gather consumer demand and trend information, thus launching personalized single products, allowing JD.com’s private labels to better meet consumer needs [11]. Additionally, Trader Joe’s is an American grocery chain with annual sales of $16.5 billion and 568 stores [12] that captures consumer demands accurately through demand forecasting, and by analyzing the innovation and relevance of existing market products, launches competitive private label products [13].

However, manufacturers, typically distant from the end market, lack direct access to real-time changes in market demand [14]. Retailers’ sharing of demand information allows upstream manufacturers to adjust their decisions, thereby increasing profitability [15]. This dynamic means manufacturers not only face competition from retailers’ private label products but also contend with the limitations of demand information.

Evaluating and balancing the choice of sales modes under conditions of informational disadvantage holds significant importance for manufacturers. Since the way manufacturers select their sales mode also affects the retailer’s revenue, retailers must carefully consider whether to share information with manufacturers under different sales modes [16]. When evaluating a retailer’s approach, manufacturers need to consider the optimal selection of sales modes and explore the possibility of a win-win outcome. Therefore, assessing the impact of retailers’ information-sharing strategies on manufacturers’ selection of sales modes is essential.

In view of this, this paper raises the following questions:

1. What is the optimal information-sharing strategy for the retailer that has introduced the private label under different sales modes?
2. Can the manufacturer and the retailer achieve a win-win outcome in sales mode selection and information-sharing strategies?
3. How does the information compensation mechanism (ICM) enable the retailer to share demand information with the manufacturer?

To solve the above problems, this study examines a supply chain with a leading manufacturer and a retailer. The retailer competes with the manufacturer’s brands in the market by selling private label products. As the Stackelberg leader, the manufacturer first determines the sales mode, after which the retailer decides whether to share demand information. Through backward induction, the equilibrium strategies of both parties can be derived. Additionally, the extended model analyzes two scenarios: the retailer acting as the leader and its private label quality being superior to that of the manufacturer’s brand. This study provides theoretical insights for promoting information-sharing and collaborative development between manufacturers and retailers, while accounting for the manufacturers’ demand information disadvantage and the introduction of private labels.

This study makes the following three contributions to the related literature. First, a new perspective on manufacturers’ sales modes selection is introduced by considering competition from self-produced private labels of equivalent and higher quality. Second, this study explores the conditions that lead to a win-win outcome for both parties in dynamic games. Third, for the first time, the information compensation mechanism (ICM) is proposed in relation to different sales modes under conditions of information asymmetry. The ICM is a contractual agreement designed to facilitate the integration of information between retailers and manufacturers.

The remainder of this study is structured in the following way. Section 2 presents a literature review. Section 3 illustrates the methods used. Section 4 examines the equilibrium results and derives the game strategies. Section 5 explores extensions to the basic model concerning the timing of the game and market demand asymmetry. Section 6 presents an extended numerical study and summarizes our findings. Finally, Section 7 offers conclusions.

## 2. Literature review

This section examines the literature by categorizing it into three main themes: sales mode selection, private labels, and demand information-sharing.

### 2.1. Sales mode selection

The selection of sales modes has been a significant topic of research over the past few decades. Several important factors influencing the choice of sales mode have been identified, including risk aversion [10], channel competition [17–19], new-version product [20], blockchain technology [21], the product demand spillover effect [22], the strategic waiting [23], two-sided platform [24], and green investment [25]. Unlike studies that focus on supply chains with information symmetry, this research explores scenarios where the retailer has a demand informational advantage. Several studies have explored how information-sharing strategies influence a manufacturer’s choice of sales mode. Liang and Ye [26] explored the interaction between an online platform’s information-sharing strategy and its sales mode selection in a supply chain with two competing manufacturers and a common online platform. Yang et al. [27] examined the selection of e-commerce sales modes and information-sharing strategies in a dual-channel supply chain. However, they have not considered the impact of competition from retailers’ private label products.

### 2.2. Private label

The penetration of private labels has been a notable trend in the retail industry. Concurrently, the private label introduction is also a crucial area in supply chain management. Multiple main factors that influence private label introduction have been analyzed, mainly focusing on the spillover effect [28], the channel encroachment [29], the cost–quality trade-off [30], and store loyalty [31]. Taking into account the sales mode selection, Cheng et al. [32] analyzed how the manufacturer and retailer compete in a dynamic manner by studying the interplay between private label and green technologies across different sales modes. They suggested that the manufacturer and retailer are inclined to adopt a reselling mode when commission costs are moderate. Hemmati et al. [33] investigated how advertising and product quality influence the selection of sales modes with private label products. Their results indicate that the dominant sales mode is strongly influenced by both advertising efforts and product quality. Zhang and Hou [34] explored how the manufacturer and the retailer choose sales modes when the retailer offers private label products. They indicated that when the commission rate is high, the manufacturer selects the reselling mode, while the retailer favors the agency selling mode. Nevertheless, these studies assume symmetric demand information and overlook the potential information advantage held by retailers.

### 2.3. Information-sharing

Information asymmetry has been widely studied in retail markets. Previous research has examined supply chain members’ information-sharing strategies from perspectives such as cost asymmetry [35], product quality asymmetry [36], and investment-level asymmetry [37]. In particular, this study focuses on demand-side information asymmetry at the supply chain’s end. Retailers, holding an informational advantage, act as key distribution channels and critical information hubs in market operations [38]. Several key factors influencing retailer information-sharing have been examined, including channel encroachment [39–44], investment strategies [45–47], pricing mechanisms [48], cap-and-trade [49], big data technology investment [50], the dual-purpose manufacturer [51], nonlinear production costs [52], trade credit [53], ordering and capacity [54]. However, the conclusions of these studies are not directly applicable to the selection of sales modes. A limited number of studies have explored how retailers’ information-sharing strategies affect manufacturers’ choice of sales mode. For instance, Zhang and Ma [14] investigated e-retailers’ motivations to share demand information with upstream competing suppliers, who can select either agency selling or reselling modes. They suggested that retailers voluntarily share information when suppliers select the reselling mode. Wei et al. [55] analyzed the optimal strategies for information-sharing through either the reselling mode alone or both the reselling and agency selling modes. Their findings suggest that when competition is sufficiently intense, both suppliers and e-retailers gain advantages by adopting dual sales modes combined with information-sharing. Chen et al. [56] examined the impact of reselling and agency selling modes on the platform’s incentives for information-sharing under both the recommendation and non-recommendation scenarios. Additionally, in the context of private label introduction, Gong et al. [15] investigated the interplay between the manufacturer’s sales format selection and the platform’s information-sharing strategies in the context of private label outsourcing. However, their study overlooked the dynamic game between these strategies and the scenario of private labels being produced in-house.

Liu et al. [57] argued that the platform always voluntarily offers demand information to the manufacturer under the agency selling mode. The issue was researched within a context where the manufacturer’s brand has a larger market share than the private label, and the ratio of their market shares exceeds the substitution coefficient between the products. Nevertheless, due to intense competition, many retailers have been motivated to improve the quality of their products [58]. As the Private Label Manufacturers Association (PLMA) claims, private label products can match the quality of manufacturer’s brand products [31]. Furthermore, a lower product substitution rate mitigates the competition between the products of both parties [59], increasing the possibility of cooperation between the manufacturer and the retailer. Therefore, a higher manufacturer’s market demand share and a lower product substitution rate influence retailers’ information-sharing strategies, which, in turn, affect manufacturers’ selection of sales modes.

The relevant literature is summarized alongside our work in Table 1.

**Table 1:** Comparison between this study and other relevant studies.

Literature	Competition: Price (P)/Quantity (Q)	Sales mode selection	Product brand categories: Private label (PL)/Manufacturer brand (MB)	Uncertain demand	Information Sharing: Exogenous (EX)/Endogenous (EN)	Produce a private label: Manufacturer (M)/ Retailer (R)	The equilibrium outcome variables	The information compensation mechanisms
Cheng et al. [32]	P	√	PL, MB	×	NAN	R	Customer sensitivity coefficient, Private label cost, Quality difference, Investment cost, Commission rate	NAN
Hemmati et al. [33]	P	√	PL, MB	×	NAN	R	Substitution rate, Quality sensitivity	NAN
Zhang and Hou. [34]	P	√	PL, MB	×	NAN	R	Sales costs, The manufacturer’s brand advantage, Commission rate	NAN
Zhang and Ma. [14]	P	√	MB	√	EN	NAN	Demand variance, Commission rate, Substitution rate	×
Chen et al. [56]	NAN	√	MB	√	EN	NAN	Information forecast accuracy, Recommendation efficiency, Commission rate	×
Yang et al. [27]	P	√	MB	√	EN	NAN	Channel substitution coefficient, Investment efficiency	×
Wei et al. [55]	P	√	MB	√	EX	NAN	Green sensitivity coefficient, Market share, Cross-price coefficient, Investment green level, Commission rate	×
Liu et al. [57]	P	√	PL, MB	√	EN	R	Commission rate	×
Gong et al. [15]	Q	√	PL, MB	√	EN	M	Information forecast accuracy, Commission rate	×
This study	Q	√	PL, MB	√	EN	R	Information forecast accuracy, Commission rate, Substitution rate	√

(√: considered; ×: not considered)

## 3. Methods

### 3.1. Notations and modeling

This paper examines a secondary supply chain involving a manufacturer and a retailer. The manufacturer sells products via two modes: reselling or agency selling (superscripts $\begin{array}{c} R\;or\;A \end{array}$, respectively), such as JD.com’s “Platform Open Plan” mode (i.e., the agency selling mode) and JD.com’s standard self-operated mode (i.e., the reselling mode). In the reselling mode, the manufacturer sells products to the retailer at a wholesale price, and the retailer sells them to consumers at a marked-up retail price. In the agency selling mode, the manufacturer sells directly to consumers and pays a commission fee to the retailer. In practice, retailers such as Amazon and JD.com also allow their cooperating manufacturers to choose between the two sales modes [60].

The retailer, with an advantage in demand information, also sells private label products that compete with the manufacturer’s brand. Walmart leverages its own demand forecasting data to guide its retail decisions. It sells products from manufacturers such as Procter & Gamble and Unilever while simultaneously promoting its private label brand Great Value. Given these dynamics, the retailer can make a decision about whether to share information or withhold it (superscripts $\begin{array}{c} S\;or\;N \end{array}$) in response to competition.

Unlike Chen et al. [56], who consider a market in which only the manufacturer’s branded products are sold, this paper focuses on a competitive market where the manufacturer’s products and the retailer’s private label products coexist. A linear inverse demand function is considered, derived from the maximization of consumer utility [29, 61].


$$U\left(q_{m},q_{r}\right)=a_{m}q_{m}+a_{r}q_{r}-\beta q_{r}q_{m}-\frac{q_{r}^{2}+q_{m}^{2}}{\text{2}}-p_{r}q_{r}-p_{m}q_{m}$$ (1)

The inverse demand functions for the manufacturer and the retailer are as below:


$$p_{m}=a_{m}-q_{m}-\beta q_{r}$$ (2)

$$p_{r}=a_{r}-q_{r}-\beta q_{m}$$ (3)

Where $a_{i}$ (i=m,r) is the market demand for both products, represents the vertical quality differential between the two brands. To capture the quality equivalence between manufacturer brands and retailer private labels, without loss of generality, it is supposed that the market demands of both are equal $a_{m}=a_{r}=a$ [26, 29]. $p_{i}$ (i=m,r) is the selling price of the manufacturer and retailer, $q_{i}$ (i=m,r) is the sales quantity of the manufacturer and retailer. β (β∈(0,1)) represents the product substitution rate between the manufacturer’s brand and the retailer’s brand, reflecting the perceptions of product variety or feature differences between the two brands.

To describe the demand uncertainty, the uncertain market size a is defined as follows:


$$a=a_{0}+\varepsilon$$ (4)

Where $a_{0}$ is the deterministic part of the demand is derived from historical data, and ε represents the random fluctuation part. The random part ε is usually distributed with a mean of 0 and a variance of $\sigma^{2}$. A larger $\sigma^{2}$ indicates greater uncertainty in market demand. To avoid extreme changes in potential market demand, it is assumed that the standard deviation of random fluctuations is less than the mean of market demand, i.e., $a_{0}>>\sigma$ [62].

Retailers have a closer connection to the consumer market and possess more information about consumer demands than manufacturers [45–47]. They can employ market data collection techniques to forecast end-market demand. The retailer’s demand prediction, denoted as f, is expressed as follows:


f=a+ς (5)

Where ς represents the error in predicting end-market demand. It is assumed that ς and ε are independent of each other, and ς follows the normal distribution with a mean of 0 and a variance of $s^{2}$.

From Eq. (5), it can be observed that if the prediction is accurate (i.e., ς=0), the predicted value equals the actual end-market demand.

Following the approach of Wei et al. [55] and Liu et al. [57], f is considered an unbiased estimator of a, and the expectation of a conditional on f is linear in f as below:


$$E\left(a|f\right)=\frac{s^{2}}{s^{2}+\sigma^{2}}a_{0}+\frac{\sigma^{2}}{s^{2}+\sigma^{2}}f$$ (6)

$$E\left({\left(f-a_{0}\right)}^{2}\right)=s^{2}+\sigma^{2}$$ (7)

Where $t=\frac{\sigma^{2}}{s^{2}+\sigma^{2}}$ is the retailer’s information forecast accuracy of demand, t∈(0,1).

Thus, Eq. (6) is transformed to:


$$E\left(a|f\right)=\left(1-t\right)a_{0}+tf$$ (8)

Assuming $\sigma^{2}$ is fixed, a lower $s^{2}$ results in a larger t, indicating a more accurate prediction of demand information. Specifically, when $s^{2}\to \infty$, t→0, the retailer’s predictions are not closely aligned with actual demand. When $s^{2}\to 0$, t→1, the retailer’s prediction is nearly identical to actual demand.

All parties in the supply chain are presumed to be risk-neutral, pursuing benefit maximization. Similarly, practices like those at JD.com include charging a fixed service fee of 0.6% on transactions [63]. The retailer’s commission rate under the agency selling mode is set as a fixed percentage of sales. Without loss of generality, the marginal production costs for both the manufacturer and the retailer are postulated to be zero, a common assumption in economic models to simplify analysis, as previously noted in the literature [15,28]. Table 2 makes a summary of the notations and definitions in the model.

**Table 2:** Indices and notations.

Indices	Description
i=m,r	Indicating the manufacturer (i=m) and the retailer (i=r)
j=A,R	Superscripts indicating the agency selling mode (j=A) and reselling mode (j=R)
e=N,S	Superscripts indicating information non-sharing (e=N) and sharing (e=S)
**Notations**	**Description**
$a_{i}$	Market demand of the manufacturer (i=m) and the retailer (i=r)
a	Market demand
$a_{0}$	Constant demand
ε	Demand random variable
ς	Market forecast variables of the retailer
$s^{2}$	Demand forecast variance
$\sigma^{2}$	Demand variance
$q_{i}$	Sales quantities of the manufacturer (i=m) and the retailer (i=r)
$p_{i}$	Sales prices of the manufacturer (i=m) and the retailer (i=r)
f	The retailer’s forecasts of market demand
w	The wholesale price of the manufacturer
t	The retailer’s information forecast accuracy
β	Product substitution rate
λ	Commission rate
η	The revenue-sharing rate
θ	The base demand ratio of the manufacturer’s brand to the retailer’s private label
$\Delta^{j}$	Distributable revenue in ICM under the agency selling mode (j=A) and the reselling mode (j=R)
$E\left(\pi_{i}^{jSC}\right)$	The expected revenues in ICM under the agency selling mode (j=A) and the reselling mode (j=R) for the manufacturer (i=m) and the retailer (i=r)
$E\left(\pi_{T}^{TC}\right)$	The total expected revenues for centralized decision-making
$E\left(\pi_{T}^{jS}\right)$	The total expected revenues for the agency selling mode (j=A) and reselling mode (j=R) under the retailer information-sharing
$E\left(\pi_{i}^{je}\right)$	The expected revenues for the manufacturer (i=m) and the retailer (i=r)

### 3.2. Game description

The information strategy employed by the retailer is a relatively long-term decision, often involving supply chain infrastructure that is costly to reverse. Similarly, the manufacturer’s choice of a sales mode is also a long-term decision and must be made before pricing decisions are set. Unlike Chen et al. [56], who assume a retailer-dominant market structure, this paper considers that the manufacturer with a market advantage acts as the Stackelberg leader, taking the lead in determining the sales mode. The retailer selling both the manufacturer’s products and its own private label products acts as the follower, possessing superior demand forecasting information and deciding the information-sharing strategies. Therefore, the events occur in the following order:

1st stage: The manufacturer selects the selling mode, either the reselling mode (denoted as R) or the agency selling mode (denoted as A).

2nd stage: The retailer chooses the demand information-sharing strategy, either to share (denoted as S) or not to share (denoted as N). The demand information will be communicated truthfully if the sharing strategy is chosen.

3rd stage: The manufacturer sets the wholesale price w under the reselling mode, or determines the sales quantities $q_{m}$ under the agency selling mode.

4th stage: The retailer sets the sales quantities $q_{m}$ and $q_{r}$ simultaneously for the manufacturer’s brand and the retailer’s private label under the reselling mode, or sets only the sales quantities $q_{r}$ for private label products under the agency selling mode.

5th stage: The consumer makes a purchase decision.

**Fig 1:**
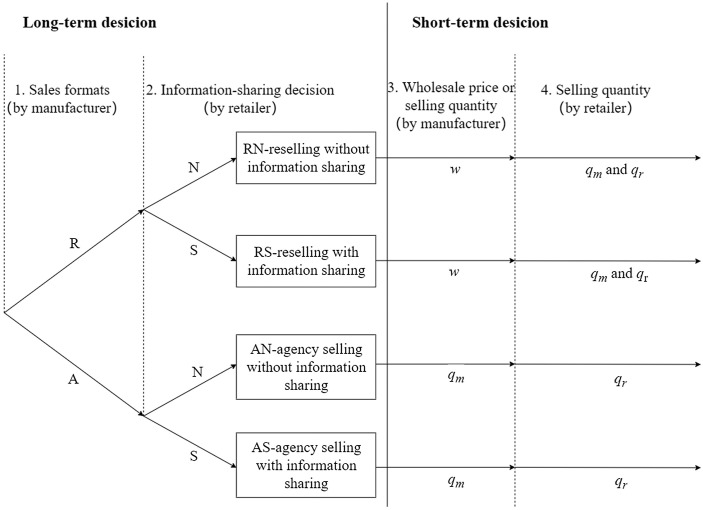
Sequence of decisions. There are two sales modes (denoted as R and A), and two information-sharing strategies (denoted as N and S). Four strategy pairs are formed: (R,N), (R,S), (A,N), and (A,S). The subgame perfect equilibrium is derived by backward induction.

The sequence of decisions is illustrated in Fig 1.

## 4. Results and analysis

In this section, the interaction between the manufacturer’s sales mode and the retailer’s information-sharing strategies is analyzed. Both the manufacturer and the retailer aim to maximize their expected revenue.

### 4.1. Equilibrium results

#### 4.1.1. Scenario (R,N): Reselling and Not sharing information.

In scenario (R,N), the manufacturer selects the reselling mode, and the retailer chooses not to share demand forecast information f. The manufacturer sells products to the retailer at a wholesale price w. Then the retailer determines the sales quantities $q_{m}$ and $q_{r}$ for the manufacturer’s brand and the retailer’s private label. The expected revenues of the manufacturer and the retailer in scenario (R,N) are as follows:


$$\underset{w}{\max}E\left(\pi_{m}^{RN}\right)=E\left(wq_{m}\right)$$ (9)

$$\underset{q_{r},q_{m}}{\max}E\left(\pi_{r}^{RN}|f\right)=E\left(\left(a-q_{r}-\beta q_{m}\right)q_{r}+\left(a-q_{m}-\beta q_{r}-w\right)q_{m}|f\right)$$ (10)

The equilibrium wholesale price and quantities under this configuration are as follows:


$$w^{RN}=\frac{\left(1-\beta \right)a_{0}}{\text{2}}$$

$$q_{m}^{RN}=\frac{2t\left(f-a_{0}\right)+a_{0}}{4\beta +4}$$

$$q_{r}^{RN}=\frac{\left(-2t+\beta +2\right)a_{0}+2tf}{4\beta +4}$$

Then, the optimal ex-ante revenues for the manufacturer and the retailer are as follows:


$$E\left(\pi_{m}^{RN}\right)=\frac{{a_{0}}^{2}\left(1-\beta \right)}{8\beta +8}$$

$$E\left(\pi_{r}^{RN}\right)=\frac{{a_{0}}^{2}\left(3\beta +5\right)+8t\sigma^{2}}{16\beta +16}$$

#### 4.1.2. Scenario (R,S): reselling and sharing information. 

In scenario (R,S), the manufacturer selects the reselling mode, and the retailer chooses to share demand forecast information f. Similarly, the expected revenues of the manufacturer and the retailer in scenario (R,S) are as follows:


$$\underset{w}{\max}E\left(\pi_{m}^{RS}|f\right)=E\left(wq_{m}|f\right)$$ (11)

$$\underset{q_{r},q_{m}}{\max}E\left(\pi_{r}^{RS}|f\right)=E\left(\left(a-q_{r}-\beta q_{m}\right)q_{r}+\left(a-q_{m}-\beta q_{r}-w\right)q_{m}|f\right)$$ (12)

The equilibrium wholesale price and quantities under this configuration are as follows:


$$w^{RS}=\frac{\left(\left(1-t\right)a_{0}+tf\right)\left(1-\beta \right)}{\text{2}}$$

$$q_{m}^{RS}=\frac{\left(1-t\right)a_{0}+tf}{4\beta +4}$$

$$q_{r}^{RS}=\frac{\left(\beta +2\right)\left(\left(1-t\right)a_{0}+tf\right)}{4\beta +4}$$

Then the optimal ex-ante revenues for the manufacturer and the retailer are as follows:


$$E\left(\pi_{m}^{RS}\right)=\frac{\left({a_{0}}^{2}+t\sigma^{2}\right)\left(1-\beta \right)}{8\left(\beta +1\right)}$$

$$E\left(\pi_{r}^{RS}\right)=\frac{\left({a_{0}}^{2}+t\sigma^{2}\right)\left(3\beta +5\right)}{16\beta +16}$$

#### 4.1.3. Scenario (A,N): agency selling and not sharing information. 

In scenario (A,N), the manufacturer selects the agency selling mode, and the retailer chooses not to share demand forecast information f. The manufacturer sells products to consumers via the retailer, and pays the retailer a commission at a rate of λ. Before the retailer sets the private label product quantities $q_{r}$, the manufacturer determines the sales quantities $q_{m}$.

The expected revenues of the manufacturer and the retailer in scenario (A,N) are as follows:


$$\underset{q_{m}}{\max}E\left(\pi_{m}^{AN}\right)=E\left(\left(1-\lambda \right)\left(a_{0}-q_{m}-\beta q_{r}\right)q_{m}\right)$$ (13)

$$\underset{q_{r}}{\max}E\left(\pi_{r}^{AN}|f\right)=E\left(\left(a-q_{r}-\beta q_{m}\right)q_{r}+\lambda \left(a-q_{m}-\beta q_{r}\right)q_{m}|f\right)$$ (14)

The equilibrium quantities under this configuration are as follows:


$$q_{m}^{AN}=\frac{a_{0}\left(2-\beta \right)}{2\left(2-\lambda \;\beta^{2}-\beta^{2}\right)}$$

$$q_{r}^{AN}=\frac{\left(4-4t\right)a_{0}+4tf-\left(\lambda +1\right)\left(\left(-2t+1\right)a_{0}+2tf\right)\beta^{2}-2a_{0}\left(\lambda +1\right)\beta }{4\left(2-\left(1+\lambda \right)\beta^{2}\right)}$$

Then, the optimal ex-ante revenues for the manufacturer and the retailer are as follows, where $\varphi_{1}=16\left(\lambda +1\mathrm{\backslash rightleft}(1-\beta \right)$:


$$E\left(\pi_{m}^{AN}\right)=\frac{\left(1-\lambda \right){a_{0}}^{2}{\left(\beta -2\right)}^{2}}{8\left(2-\left(1+\lambda \right)\beta^{2}\right)}$$

$$E\left(\pi_{r}^{AN}\right)=\frac{{a_{0}}^{2}\left({\left(\lambda +1\right)}^{2}\beta^{4}+\left(12\lambda^{2}+16\lambda +4\right)\beta^{3}+\left(-12\lambda^{2}-20\lambda -4\right)\beta^{2}+\varphi_{1}\right)}{16{\left(-2+\beta^{2}\left(\lambda +1\right)\right)}^{2}}+\frac{t\sigma^{2}}{\text{4}}$$

#### 4.1.4. Scenario (A,S): Agency selling and Sharing information. 

In scenario (A,S), the manufacturer selects the agency selling mode, and the retailer chooses to share demand forecast information. Similarly, the expected revenues of the manufacturer and the retailer in scenario (A,S) are as follows:


$$\underset{q_{m}}{\max}E\left(\pi_{m}^{AS}|f\right)=E\left(\left(1-\lambda \right)\left(a-q_{m}-\beta q_{r}\right)q_{m}|f\right)$$ (15)

$$\underset{q_{r}}{\max}E\left(\pi_{r}^{AS}|f\right)=E\left(\left(a-q_{r}-\beta q_{m}\right)q_{r}+\lambda \left(a-q_{m}-\beta q_{r}\right)q_{m}|f\right)$$ (16)

The equilibrium quantities under this configuration are as follows:


$$q_{m}^{AS}=\frac{\left(\left(1-t\right)a_{0}+tf\right)\left(2-\beta \right)}{2\left(2-\lambda \;\beta^{2}+\beta^{2}\right)}$$

$$q_{r}^{AS}=\frac{\left(4-\beta^{2}\left(\lambda +1\right)-\left(2\lambda +2\right)\beta \right)\left(\left(1-t\right)a_{0}+tf\right)}{4\left(2-\left(1+\lambda \right)\beta^{2}\right)}$$

Then, the optimal ex-ante revenues for the manufacturer and the retailer are as below:


$$E\left(\pi_{m}^{AS}\right)=\frac{\left(1-\lambda \right)\left({a_{0}}^{2}+t\sigma^{2}\right){\left(2-\beta \right)}^{2}}{8\left(2-\left(\lambda +1\right)\beta^{2}\right)}$$

$$E\left(\pi_{r}^{AS}\right)=\frac{\left({a_{0}}^{2}+t\sigma^{2}\right)\left({\left(\lambda +1\right)}^{2}\beta^{4}+\left(12\lambda^{2}+16\lambda +4\right)\beta^{3}+\left(-12\lambda^{2}-20\lambda -4\right)\beta^{2}+\varphi_{1}\right)}{16{\left(2-\left(\lambda +1\right)\beta^{2}\right)}^{2}}$$

The equilibrium solutions for the four scenarios are demonstrated in Table 3.

**Table 3:** The optimal equilibrium outcomes of four scenarios.

The optimal equilibrium outcomes	
(R,N)	$w^{RN*}=\frac{\left(1-\beta \right)a_{0}}{\text{2}}$, $q_{m}^{RN*}=\frac{2t\left(f-a_{0}\right)+a_{0}}{4\beta +4}$, $q_{r}^{RN*}=\frac{\left(-2t+\beta +2\right)a_{0}+2tf}{4\beta +4}$, $E\left(\pi_{m}^{RN}\right)=\frac{{a_{0}}^{2}\left(1-\beta \right)}{8\beta +8}$, $E\left(\pi_{r}^{RN}\right)=\frac{{a_{0}}^{2}\left(3\beta +5\right)+8t\sigma^{2}}{16\beta +16}$.
(R,S)	$w^{RS*}=\frac{\left(\left(1-t\right)a_{0}+tf\mathrm{\backslash rightleft}(1-\beta \right)}{\text{2}}$, $q_{m}^{RS*}=\frac{\left(1-t\right)a_{0}+tf}{4\beta +4}$, $q_{r}^{RS*}=\frac{\left(\beta +2\mathrm{\backslash rightleft}(\left(1-t\right)a_{0}+tf\right)}{4\beta +4}$ $E\left(\pi_{r}^{RS}\right)=\frac{\left({a_{0}}^{2}+t\sigma^{2}\mathrm{\backslash rightleft}(3\beta +5\right)}{16\left(1+\beta \right)}$, $E\left(\pi_{m}^{RS}\right)=\frac{\left({a_{0}}^{2}+t\sigma^{2}\mathrm{\backslash rightleft}(1-\beta \right)}{8\left(1+\beta \right)}$
(A,N)	$q_{r}^{AN*}=\frac{\left(4-4t\right)a_{0}+4tf-\left(\lambda +1\mathrm{\backslash rightleft}(\left(-2t+1\right)a_{0}+2tf\right)\beta^{2}+2a_{0}\left(\lambda +1\right)\beta }{8-\left(4\lambda +4\right)\beta^{2}}$, $q_{m}^{AN*}=\frac{a_{0}\left(2-\beta \right)}{2\left(2-\lambda \;\beta^{2}-\beta^{2}\right)}$, $E\left(\pi_{m}^{AN}\right)=\frac{\left(1-\lambda \right){a_{0}}^{2}{\left(\beta -2\right)}^{2}}{16-\left(8\lambda +8\right)\beta^{2}}$, $E\left(\pi_{r}^{AN}\right)=\frac{{a_{0}}^{2}\left({\left(\lambda +1\right)}^{2}\beta^{4}+\left(12\lambda^{2}+16\lambda +4\right)\beta^{3}+\left(-12\lambda^{2}-20\lambda -4\right)\beta^{2}+\varphi_{1}\right)}{16{\left(-2+\beta^{2}\left(\lambda +1\right)\right)}^{2}}+\frac{t\sigma^{2}}{\text{4}}$.
(A,S)	$q_{m}^{AS*}=\frac{\left(\left(1-t\right)a_{0}+tf\mathrm{\backslash rightleft}(2-\beta \right)}{2\left(2-\lambda \;\beta^{2}+\beta^{2}\right)}$, $E\left(\pi_{m}^{AS}\right)=\frac{\left(1-\lambda \mathrm{\backslash rightleft}({a_{0}}^{2}+t\sigma^{2}\right){\left(\beta -2\right)}^{2}}{16-\left(8\lambda +8\right)\beta^{2}}$, $E\left(\pi_{m}^{AS}\right)=\frac{\left(1-\lambda \mathrm{\backslash rightleft}({a_{0}}^{2}+t\sigma^{2}\right){\left(\beta -2\right)}^{2}}{16-\left(8\lambda +8\right)\beta^{2}}$, $E\left(\pi_{r}^{AS}\right)=\frac{\left({a_{0}}^{2}+t\sigma^{2}\mathrm{\backslash rightleft}({\left(\lambda +1\right)}^{2}\beta^{4}+\left(12\lambda^{2}+16\lambda +4\right)\beta^{3}+\left(-12\lambda^{2}-20\lambda -4\right)\beta^{2}+\varphi_{1}\right)}{16{\left(2-\left(\lambda +1\right)\beta^{2}\right)}^{2}}$.

Retailers are less likely to impose excessively high consignment commission rates, as maintaining long-term partnerships with manufacturers is a key strategic consideration. It can be assumed that when $\sqrt{3}-1<\beta <1$, $0<\lambda <\min\left\{\lambda_{1},1\right\}$, where $\lambda_{1}=\frac{-\beta^{2}-2\beta +4}{\beta^{2}+2\beta }$.

### 4.2. Strategy analysis

#### 4.2.1. Information-sharing strategies in the reselling mode.

The following proposition is derived by comparing the expected revenues when the retailer shares or does not share forecast information in the reselling mode. All proofs of propositions and corollaries are provided in the S1 File.

**Proposition 1:** The retailer chooses not to share market demand forecast information when the manufacturer chooses the reselling mode, i.e., $E\left(\pi_{r}^{RS}\right)<E\left(\pi_{r}^{RN}\right)$.

Proposition 1 proposes that when the manufacturer selects the reselling mode, the retailer tends to keep information private. This behavior exacerbates the double marginalization effect, thereby reducing the downstream retailer’s profitability [57].

#### 4.2.2. Information-sharing strategies in the agency selling mode.

For clarity and conciseness in illustrating the sub-game equilibrium results, the agency commission rate threshold is defined as follows:


$$\lambda_{2}=\frac{3\beta^{3}-2\beta^{2}-2\sqrt{7\beta^{4}-4\beta^{3}-3\beta^{2}-4\beta +4}-2\beta +4}{3\beta^{2}\left(2-\beta \right)}$$

The following proposition is derived.

**Proposition 2:** When 0<β<2/3 and $\lambda_{2}<\lambda <1$, the retailer chooses to share market demand forecast information with the manufacturer in the agency selling mode, i.e., $E\left(\pi_{r}^{AS}\right)>E\left(\pi_{r}^{AN}\right)$; otherwise, the retailer chooses not to share it.

Under the agency selling mode, the retailer’s expected revenue includes both the commission from the manufacturer and earnings from selling private label products. The competition between the manufacturer and the retailer is moderated by the commission rate, and the adverse effects of double marginalization at a specific product substitution rate are reduced. Additionally, the commission rate threshold $\lambda_{2}$ increases with the increase of β ($\partial \lambda_{2}/\partial \beta >0$), which also reflects the influence of the product substitution coefficient on the retailer’s decision to share information under the agency selling mode. When product substitution is low and the commission rate exceeds a certain threshold, the retailer expects revenue increases to be greater than competitive losses from sharing information under the agency selling mode. Therefore, the retailer is more inclined to share market demand forecast information with the manufacturer. However, under other conditions, the retailer will not proactively share information with the manufacturer. This finding differs from the research by Liu et al. [57], who suggested that the retailer always chooses to share information with the manufacturer under the agency selling mode.

#### 4.2.3. Manufacturer’s sales mode selection.

The retailer’s expected revenue under different sales modes is compared based on the subgames in Propositions 1 and 2. From this comparison, the manufacturer’s optimal sales mode selection and the subgame perfect equilibrium are derived.

For clarity and conciseness in presenting the final equilibrium results, six thresholds are defined:


$$\lambda_{3}=\frac{1-\beta^{2}+\beta }{\beta^{3}-2\beta^{2}+2}$$

$$\lambda_{4}=\frac{\beta^{3}-5\beta^{2}+2\beta +6}{3\beta^{3}-7\beta^{2}+8}$$

$$t_{1}=\frac{2{a_{0}}^{2}\left(\lambda \;\beta^{3}-2\lambda \;\beta^{2}+\beta^{2}-\beta +2\lambda -1\right)}{\left(\beta +1\right)\left(1-\lambda \right)\sigma^{2}{\left(\beta -2\right)}^{2}}$$

$0<\beta_{1}<2/3$ is a solution of $\lambda_{3}-\lambda_{2}=0$

$0<\beta_{2}<2/3$ is a solution of $\lambda_{4}-\lambda_{2}=0$

$\sqrt{3}-1<\beta_{3}<1$ is a solution of $\lambda_{3}-\lambda_{1}=0$

**Proposition 3:** The manufacturer’s sales mode and the subgame perfect equilibrium are as follows:

(1) When $0<\beta <\beta_{1}$ and $0<\lambda <\lambda_{2}$, the manufacturer selects the agency selling mode, i.e., $E\left(\pi_{m}^{RN}\right)<E\left(\pi_{m}^{AN}\right)$. The subgame perfect equilibrium is (A,N). When (i) $0<\beta <\beta_{1}$ and $\lambda_{2}\leq \lambda <\lambda_{3}$, or (ii) $0<\beta <\beta_{1}$, $\lambda_{3}\leq \lambda <\lambda_{4}$ and $t_{1}<t<1$, the manufacturer selects the agency selling mode, i.e., $E\left(\pi_{m}^{RN}\right)$$<E\left(\pi_{m}^{AS}\right)$. The subgame perfect equilibrium is (A,S). When (i) $0<\beta <\beta_{1}$, $\lambda_{3}\leq \lambda <\lambda_{4}$ and $0<t\leq \min\left\{t_{1},1\right\}$, or (ii) $0<\beta <\beta_{1}$ and $\lambda_{4}\leq \lambda <1$, the manufacturer selects the reselling mode, i.e., $E\left(\pi_{m}^{RN}\right)\geq E\left(\pi_{m}^{AS}\right)$. The subgame perfect equilibrium is (R,N).
(2) When $\beta_{1}\leq \beta <\beta_{2}$ and $0<\lambda <\lambda_{3}$, the manufacturer selects the agency selling mode, i.e., $E\left(\pi_{m}^{RN}\right)<E\left(\pi_{m}^{AN}\right)$. The subgame perfect equilibrium is (A,N). When $\beta_{1}\leq \beta <\beta_{2}$ and $\lambda_{3}\leq \lambda \leq \lambda_{2}$, the manufacturer selects the agency selling mode, i.e., $E\left(\pi_{m}^{RN}\right)\geq E\left(\pi_{m}^{AN}\right)$. The subgame perfect equilibrium is (R,N). When $\beta_{1}\leq \beta <\beta_{2}$, $\lambda_{2}<\lambda <\lambda_{4}$ and $t_{1}<t<1$, the manufacturer selects the agency selling mode, i.e., $E\left(\pi_{m}^{RN}\right)<E\left(\pi_{m}^{AS}\right)$. The subgame perfect equilibrium is (A,S). When (i) $\beta_{1}\leq \beta <\beta_{2}$, $\lambda_{2}<\lambda <\lambda_{4}$ and 0<t≤$\min\left\{t_{1},1\right\}$, or (ii) $\beta_{1}\leq \beta <\beta_{2}$ and $\lambda_{4}\leq \lambda <1$, the manufacturer selects the reselling mode, i.e., $E\left(\pi_{m}^{RN}\right)\geq E\left(\pi_{m}^{AS}\right)$. The subgame perfect equilibrium is (R,N).
(3) When $\beta_{2}\leq \beta <2/3$ and $0<\lambda <\lambda_{3}$, the manufacturer selects the agency selling mode, i.e., $E\left(\pi_{m}^{RN}\right)<E\left(\pi_{m}^{AN}\right)$. The subgame perfect equilibrium is (A,N). When $\beta_{2}\leq \beta <2/3$ and $\lambda_{3}\leq \lambda \leq \lambda_{2}$, the manufacturer selects the reselling mode, i.e., $E\left(\pi_{m}^{RN}\right)\geq E\left(\pi_{m}^{AN}\right)$. The subgame perfect equilibrium is (R,N). When $\beta_{2}\leq \beta <2/3$ and $\lambda_{2}<\lambda <1$, the manufacturer selects the reselling mode, i.e., $E\left(\pi_{m}^{RN}\right)\geq E\left(\pi_{m}^{AS}\right)$. The subgame perfect equilibrium is (R,N).
(4) When $2/3\leq \beta <\beta_{3}$ and $0<\lambda <\lambda_{3}$, the manufacturer selects the agency selling mode, i.e., $E\left(\pi_{m}^{RN}\right)<E\left(\pi_{m}^{AN}\right)$. The subgame perfect equilibrium is (A,N). When (i) $2/3\leq \beta <\sqrt{3}-1$ and $\lambda_{3}\leq \lambda <1$, or (ii) $\sqrt{3}-1\leq \beta <\beta_{3}$ and $\lambda_{3}\leq \lambda <\lambda_{1}$ the manufacturer selects the reselling mode, i.e., $E\left(\pi_{m}^{RN}\right)\geq$$E\left(\pi_{m}^{AN}\right)$. The subgame perfect equilibrium is (R,N).
(5) When $\beta_{3}\leq \beta <1$ and $0<\lambda <\lambda_{1}$, the manufacturer selects the agency selling mode, i.e., $E\left(\pi_{m}^{RN}\right)<E\left(\pi_{m}^{AN}\right)$. The subgame perfect equilibrium is (A,N).

**Fig 2:**
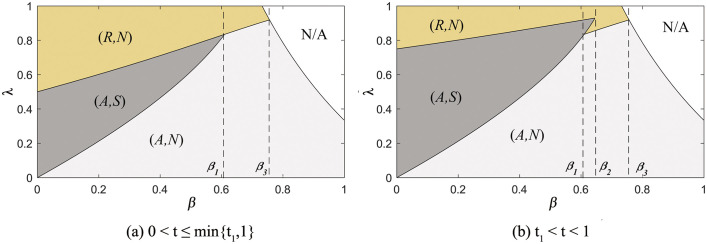
The subgame perfect equilibrium.

The results of Proposition 3 are presented in Fig 2, showing how the commission rate, product substitution rate, and the retailer’s information forecast accuracy influence equilibrium strategies.

When product competition is fierce or the commission rate is low (i.e., (i) 2/3≤β<1 or (ii) 0<β<2/3 and $0<\lambda \leq \lambda_{2}$), the retailer will withhold the information, regardless of the manufacturer’s sales mode selection. In this scenario, the manufacturer’s selection of sales mode depends solely on the commission rate, with excessively high commission rates driving the manufacturer to select the reselling mode (i.e., $\lambda_{3}\leq \lambda <1$). When the product substitution rate is low, and the commission rate is high (i.e., 0<β<2/3 and $\lambda_{2}<\lambda <1$), the retailer will share demand information only if the manufacturer selects agency selling. At this point, the manufacturer must compare the expected revenue from agency selling (where the retailer shares the information) with that from reselling (where the retailer does not share the information).

Figs 2(a) and 2(b) demonstrate that the manufacturer will consistently choose the reselling mode when the commission rate is excessively high (i.e., $\lambda \geq \lambda_{4}$). This decision is independent of the product substitution rates and the retailer’s information forecast accuracy. In practice, this reflects real-world scenarios where a high commission rate makes agency selling unprofitable for the manufacturer. When the commission rate is moderate (i.e., $\max\left\{\lambda_{3},\lambda_{2}\right\}<\lambda <\lambda_{4}$), and both demand variance and the retailer’s information forecast accuracy are high (i.e., $\sigma^{2}>$$\frac{2a_{0}^{2}\left(\lambda \;\beta^{3}+\left(1-2\lambda \right)\beta^{2}-\beta +2\lambda -1\right)}{\left(1+\beta \mathrm{\backslash rightleft}(1-\lambda \right){\left(-2+\beta \right)}^{2}}$ and $t_{1}<t<1$), the manufacturer selects the agency selling mode. Because greater information forecast accuracy increases the benefits of information-sharing for the manufacturer, it creates more incentives for manufacturers to select the agency selling mode. Conversely, if the retailer’s information forecast accuracy or demand variance is low (i.e., $0<t\leq t_{1}$, or $\sigma^{2}\leq$$\frac{2a_{0}^{2}\left(\lambda \;\beta^{3}+\left(1-2\lambda \right)\beta^{2}-\beta +2\lambda -1\right)}{\left(1+\beta \mathrm{\backslash rightleft}(1-\lambda \right){\left(-2+\beta \right)}^{2}}$, which means $t_{1}>1$), the manufacturer selects the reselling mode. In this case, the manufacturer’s increased expected revenue from information-sharing does not outweigh the losses associated with the agency selling mode.

This result contrasts with the conclusions of Liu et al. [57], who suggest that the equilibrium outcome is the (A,S) scenario only when the commission rate remains below a specific threshold. Our research aligns with business practice. In reality, when product substitution is high, retailers may choose to withhold demand information, even if the manufacturer selects the agency selling mode. Retailers often view this information as a competitive advantage. Amazon leverages consumer data from its extensive network of third-party sellers to develop its own private label products, which have already presented a significant competitive challenge to similar products [64].

The manufacturer’s choice of agency selling mode can facilitate the integration of supply chain information. For example, Terapeak, a data analysis tool launched by eBay, can provide third-party sellers with free services such as market trend analysis and predictions of seasonal consumer trends on a certain scale [65].

The influences of the retailer’s demand information-sharing strategies on the manufacturer’s expected revenue under the two sales modes are outlined as follows:

**Proposition 4:** The manufacturer’s expected revenue increases in both reselling and agency selling modes when the retailer shares information, i.e., $E\left(\pi_{m}^{RS}\right)>E\left(\pi_{m}^{RN}\right)$, $E\left(\pi_{m}^{AS}\right)>E\left(\pi_{m}^{AN}\right)$.

Proposition 4 indicates that, whether the manufacturer selects the reselling or agency selling mode, the retailer’s information-sharing contributes to an increase in the manufacturer’s expected revenue. When the retailer shares market forecast information, the retailer’s information advantage is diminished. This enables the manufacturer to make more informed decisions concerning wholesale prices or quantities, leading to greater revenue compared to when information is withheld.

In addition, in conjunction with the conclusions of Propositions 3 and 4, the win-win outcome can be deduced in Proposition 5. For clarity and conciseness in presenting the results, three thresholds are defined:


$$\lambda_{5}=\frac{\left(\beta^{2}-\beta -2\right)\sqrt{6\beta^{4}-2\beta^{3}-4\beta^{2}-4\beta +4}-\beta^{5}+2\beta^{4}+2\beta^{3}-4\beta^{2}+4}{\beta^{2}\left(\beta^{3}-4\beta^{2}+6\right)}$$

$$t_{2}=\frac{2\left({\left(\lambda +1\right)}^{2}\beta^{5}+\left(-4\lambda^{2}-4\lambda \right)\beta^{4}+\left(-4\lambda -6\right)\beta^{3}+\left(6\lambda^{2}+8\lambda \right)\beta^{2}+6\beta -8\lambda +2\right)a_{0}^{2}}{\left({\left(\lambda +1\right)}^{2}\beta^{5}+\left(5\lambda^{2}+2\lambda -3\right)\beta^{4}-4\beta^{3}\lambda +\left(-12\lambda^{2}-4\lambda +12\right)\beta^{2}+16\lambda -16\right)\sigma^{2}}$$

$\beta_{4}$ is a solution of $\beta^{6}-3\beta^{5}+\beta^{4}+5\beta^{3}-6\beta^{2}-2\beta +2=0$

**Proposition 5:** When $0<\beta <\beta_{4}$, $\lambda_{5}<\lambda <\lambda_{3}$ and $0<t<\min\left\{t_{2},1\right\}$, a win-win outcome for both retailer and manufacturer can be achieved under the agency selling mode.

According to Proposition 5, when the substitution rate between the manufacturer’s and the retailer’s private label products is low (i.e., $0<\beta <\beta_{4}$), the commission rate is within a relatively low range (i.e., $\lambda_{5}<\lambda <\lambda_{3}$), and the retailer’s information forecast accuracy falls within the interval $0<t<\min\left\{t_{2},1\right\}$, both the manufacturer and the retailer can achieve a win-win outcome under the agency mode.

When competition between the two parties is not intense (i.e., 0<β<2/3), the retailer will share demand information once the commission rate is within the range $\lambda_{2}<\lambda <1$. This allows the manufacturer to benefit from choosing the agency selling mode even at slightly higher product substitution rates and slightly higher commission rates (i.e., $0<\beta <\beta_{1}$ and $\lambda_{2}\leq \lambda <\lambda_{3}$).

When weighing sales mode selections, the retailer must consider not only its own information-sharing strategy but also the product substitution rate, the commission rate, and its information forecast accuracy. Specifically, when the product substitution rate is low (i.e., $0<\beta <\beta_{1}$ and $\lambda_{2}\leq \lambda <\lambda_{3}$), market competition is relatively mild. Even if the retailer loses its information advantage, it can still benefit from the agency selling mode by charging a slightly higher commission rate (i.e., $\lambda_{5}<\lambda <\lambda_{3}$), provided that the retailer’s information forecast accuracy lies within the interval $0<t<\min\left\{t_{2},1\right\}$. Overall, this implies that the manufacturer and the retailer can achieve mutual gains through the agency selling mode.

**Fig 3:**
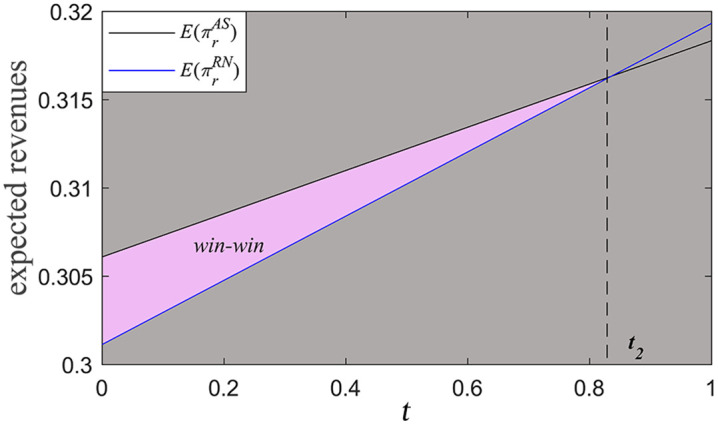
Win-win area ($a_{0}=1$, σ=0.2, β=0.1, λ=0.35).

Fig 3 illustrates the win-win area for the manufacturer and the retailer under the agency selling mode. When $\lambda =0.35\in \left(\lambda_{5},\lambda_{3}\right)$, $t_{2}\approx 0.835$. When $0<t<t_{2}$ and $\lambda_{5}<\lambda <\lambda_{3}$, $E\left(\pi_{r}^{AS}\right)>E\left(\pi_{r}^{RN}\right)$. In this case, the retailer’s expected revenue from information-sharing under the agency selling mode exceeds that under the reselling mode (i.e., $E\left(\pi_{r}^{AS}\right)>E\left(\pi_{r}^{RN}\right)>E\left(\pi_{r}^{RS}\right)$). At the same time, when $0<\lambda <\lambda_{3}$, the manufacturer’s expected revenue from information-sharing under the agency selling mode is also higher than that under the reselling mode (i.e., $E\left(\pi_{m}^{AS}\right)>E\left(\pi_{m}^{RS}\right)$). Therefore, consistent with the analysis in Proposition 3, a win-win area exists in which the manufacturer selects the agency selling mode while the retailer chooses to share demand forecast information.

### 4.3. Mechanism design

According to Propositions 1 and 4, when the manufacturer selects the reselling mode under certain conditions, the retailer’s information-sharing behavior will consistently improve the manufacturer’s revenue and reduce the retailer’s expected revenue. Consequently, the retailer has no incentive to share its private forecast information.

Based on Propositions 2 and 4, when the manufacturer selects the agency selling mode, it still benefits from the retailer’s information-sharing. However, in this case, the retailer will only share forecast information with the manufacturer under certain conditions.

Therefore, in contrast to Chen et al. [56], who focus solely on the value of information-sharing for supply chain members, this paper introduces an Information Compensation Mechanism (ICM) designed to achieve mutual benefit for both parties. Under the reselling mode, the manufacturer’s and retailer’s expected revenues within the ICM framework are denoted as $E\left(\pi_{m}^{RSC}\right)$ and $E\left(\pi_{r}^{RSC}\right)$, respectively. Under the agency selling mode, when conditions (i) 2/3≤β<1 and $0<\lambda \leq \lambda_{1}$; or (ii) 0<β<2/3 and $0<\lambda \leq \lambda_{2}$ are satisfied, their corresponding expected revenues are denoted as $E\left(\pi_{m}^{ASC}\right)$ and $E\left(\pi_{r}^{ASC}\right)$.

Following the core logic of Incentive Compatibility Theory, the retailer’s expected revenue with information-sharing should be greater than that without information-sharing, while the manufacturer’s expected revenue should be no less than that achieved under the information-sharing scenario. Accordingly, the information compensation mechanism must satisfy the following revenue constraints:


$$\{\begin{array}{c} {}*20cE\left(\pi_{r}^{RSC}\right)>E\left(\pi_{r}^{RN}\right)\\ E\left(\pi_{m}^{RSC}\right)\geq E\left(\pi_{m}^{RS}\right) \end{array}$$ (17)

$$\{\begin{array}{c} {}*20cE\left(\pi_{r}^{ASC}\right)>E\left(\pi_{r}^{AN}\right)\\ E\left(\pi_{m}^{ASC}\right)\geq E\left(\pi_{m}^{AS}\right) \end{array}$$ (18)

Let the total expected revenue of the supply chain under centralized decision-making be denoted as $E\left(\pi_{T}^{TC}\right)$. Under decentralized decision-making, the total expected revenues of the supply chain in the reselling and agency selling modes are represented by $E\left(\pi_{T}^{RS}\right)$ and $E\left(\pi_{T}^{AS}\right)$, respectively. The corresponding distributable information compensation revenues under the two sales modes are $\Delta^{R}$ and $\Delta^{A}$, where $\Delta^{R}=E\left(\pi_{T}^{TC}\right)-E\left(\pi_{T}^{RS}\right)$, $\Delta^{A}=E\left(\pi_{T}^{TC}\right)-E\left(\pi_{T}^{AS}\right)$. When $\Delta^{R}>0$ and $\Delta^{A}>0$, the feasibility of allocating the information compensation revenue is ensured.

Consider the ICM that allocates the increased total revenue between the manufacturer and retailer when the ICM is agreed upon. The ICM specifies a revenue-sharing rate η, where the retailer receives a portion of η, and the manufacturer receives the remaining 1−η, with η∈(0,1). Therefore, under the two different sales modes, the manufacturer’s and retailer’s expected revenues after applying the ICM can be expressed as follows:


$$\{\begin{array}{c} {}*20lE\left(\pi_{m}^{RSC}\right)=(1-\eta )\Delta^{R}+E\left(\pi_{m}^{RS}\right)\\ E\left(\pi_{r}^{RSC}\right)=\eta \Delta^{R}+E\left(\pi_{r}^{RS}\right) \end{array}$$ (19)

$$\{\begin{array}{c} {}*20lE\left(\pi_{m}^{ASC}\right)=(1-\eta )\Delta^{A}+E\left(\pi_{m}^{AS}\right)\\ E\left(\pi_{r}^{ASC}\right)=\eta \Delta^{A}+E\left(\pi_{r}^{AS}\right) \end{array}$$ (20)

For clarity and conciseness in presenting the results, six thresholds are defined:


$$\eta_{1}=\frac{3t\sigma^{2}}{t\sigma^{2}+{a_{0}}^{2}}$$

$$t_{3}=\frac{{a_{0}}^{2}}{2\sigma^{2}}$$

$$\lambda_{6}=\frac{\beta \left(4-\beta^{2}-\beta \right)}{3\beta^{3}-5\beta^{2}+4}$$

$$\eta_{2}=\frac{\left(3{\left(\lambda +1\right)}^{2}\beta^{3}+\left(-6\lambda^{2}-4\lambda +2\right)\beta^{2}+\left(-4\lambda -8\right)\beta +8\lambda \right)\left(\beta +1\right)\left(\beta -2\right)t\sigma^{2}}{\beta^{2}\left(\left(-3\lambda^{2}-2\lambda +1\right)\beta^{3}+\left(\lambda^{2}-2\lambda -3\right)\beta^{2}+\left(8\lambda -4\right)\beta +4\lambda^{2}-8\lambda +8\right)\left(t\sigma^{2}+{a_{0}}^{2}\right)}$$

$$t_{4}=\frac{{a_{0}}^{2}\beta^{2}\left(-3\beta^{3}\lambda^{2}-2\beta^{3}\lambda +\beta^{2}\lambda^{2}+\beta^{3}-2\beta^{2}\lambda -3\beta^{2}+8\beta \lambda +4\lambda^{2}-4\beta -8\lambda +8\right)}{2\sigma^{2}\left(\beta^{2}\lambda +\beta^{2}-2\right)\left(3\beta^{3}\lambda +\beta^{3}-5\beta^{2}\lambda +\beta^{2}-4\beta +4\lambda \right)}$$

$\beta_{5}\in \left(\beta_{1},1\right)$ is a solution of $\lambda_{1}-\lambda_{6}=0$

The following proposition is derived:

**Proposition 6:** (1) In the reselling mode, the ICM allows the retailer to share information if $0<t<\min\{1,t_{3}\}$ and $\eta_{1}<\eta <1$. (2) In the agency selling mode, the ICM allows the retailer to share information under the following conditions: (i) $0<\beta <\beta_{3}$, $0<\lambda <\lambda_{6}$, $0<t<\min\left\{1,t_{4}\right\}$ and $\eta_{2}<\eta <1$; or (ii) $\beta_{3}\leq \beta <1$, 0<λ<min$\left\{\lambda_{1},\lambda_{6}\right\}$, $0<t<\min\left\{1,t_{4}\right\}$ and $\eta_{2}<\eta <1$; or (iii) 0<β<2/3, $\lambda_{6}<\lambda \leq \lambda_{2}$ and $\eta_{2}<\eta <1$; or (iv) $2/3\leq \beta <\beta_{3}$, $\lambda_{6}$$<\lambda <\lambda_{3}$ and $\eta_{2}<\eta <1$; or (v) $\beta_{3}\leq$$\beta <\beta_{5}$, $\lambda_{6}<\lambda <\lambda_{1}$ and $\eta_{2}<\eta <1$.

According to Proposition 6, in the reselling mode, when the demand variance is low and the revenue-sharing rate is high (i.e., $\sigma^{2}\leq a_{0}^{2}/2$ which means $t_{3}>1$, and $\eta_{1}<\eta <1$), the retailer will consent to collaborate with the manufacturer and share the demand information. A smaller market demand variance leads to less loss for the retailer from sharing information. When the revenue-sharing rate is high, the expected revenue increase under the ICM outweighs the retailer’s loss from information-sharing.

However, when the market demand variance is high and the retailer’s information forecast accuracy is high (i.e., $a_{0}^{2}/2<\sigma^{2}<a_{0}^{2}$ which means $t_{3}\in \left(0,1\right)$, and $t\geq t_{3}$), sharing information would lead to significant losses for the retailer. In this scenario, the revenue gained from cooperating with the manufacturer is insufficient to cover the losses caused by sharing information, so the retailer will withhold information to maintain its advantage.

Conversely, when the market demand variance is high and the retailer’s information forecast accuracy is low (i.e., $a_{0}^{2}/2<\sigma^{2}<a_{0}^{2}$ and $0<t<t_{3}$), the loss from sharing information is reduced. If the revenue-sharing rate is high (i.e., $\eta_{1}<\eta <1$), the increase in expected revenue from the ICM will exceed the loss from sharing information, prompting the retailer to share information and cooperate with the manufacturer.

In the agency selling mode, when the commission rate and the market demand variance threshold are low (i.e., $0<\lambda <\lambda_{7}$ and $0<t_{4}<1$, where $\lambda_{7}=$$\frac{\varphi_{2}-2\sqrt{4\beta^{10}-16\beta^{9}-\beta^{8}+70\beta^{7}-52\beta^{6}-94\beta^{5}+105\beta^{4}+40\beta^{3}-72\beta^{2}+16}}{\beta^{2}\left(9\beta^{3}-11\beta^{2}+4\right)}$ $<\lambda_{2}$, where $\varphi_{2}=-5\beta^{5}+3\beta^{4}+14\beta^{3}-18\beta^{2}+8$), and the retailer’s information forecast accuracy is high (i.e., $t_{4}\leq t<1$), its expected revenue from sharing information with the manufacturer will be less than that if it does not share. In this scenario, the retailer chooses not to cooperate with the manufacturer. Conversely, if the retailer’s information forecast accuracy is low (i.e., $0<t<t_{4}$) and the revenue-sharing rate is high (i.e., $\eta_{2}<\eta <1$), the retailer will accept the ICM.

Additionally, when the commission rate is moderate (i.e., $\lambda_{7}\leq \lambda <\lambda_{6}$, which means $t_{4}>1$) or the commission rate is low and the market demand variance threshold is high (i.e., $0<\lambda <\lambda_{7}$ and $t_{4}>1$), the retailer chooses to share information and cooperate with the manufacturer if the revenue-sharing rate is high (i.e., $\eta_{2}<\eta <1$), regardless of the retailer’s information forecast accuracy.

Furthermore, when (i) 0<β<2/3 and $\lambda_{6}<\lambda \leq \lambda_{2}$; (ii) $2/3\leq \beta <\beta_{3}$ and $\lambda_{6}$$<\lambda <\lambda_{3}$; or (iii) $\beta_{3}\leq$$\beta <\beta_{5}$, $\lambda_{6}<\lambda <\lambda_{1}$, a revenue-sharing rate threshold $\eta_{2}$ (i.e., $0<\eta_{2}\leq 1/2$) exists. When $\eta_{2}<\eta <1$, the retailer chooses to share information and cooperate with the manufacturer. Notably, in this scenario, a higher commission rate corresponds to a lower revenue-sharing rate threshold.

From the viewpoint of the manufacturer, low demand variance and the retailer’s low information forecast accuracy promote cooperation under the reselling mode. In the agency selling mode, in addition to these factors, a higher commission rate also facilitates cooperation.

## 5. Extensions

### 5.1. Long-term decision sequence

In the basic mode, the manufacturer initially chooses the sales mode, after which the retailer determines the information-sharing strategy. However, in practice, there are situations in operations management where the retailer decides in advance whether to share forecast information. For example, the cooperation between JD.com and ZTE has achieved Customer-to-Manufacturer (C2M) reverse customization [66]. Therefore, the model extension in this study considers scenarios in the long-term decision-making where the retailer’s demand information sharing strategies precede the manufacturer’s selection of sales modes. As in the previous analysis, the retailer first determines whether to share information or not (S or N), and the manufacturer subsequently chooses between reselling or agency selling (R or A). To distinguish from the basic mode, according to the sequence of the game, the pure strategy combinations are represented as (S,R), (S,A), (N,R), (N,A), with both parties’ expected revenues remaining unchanged.

The manufacturer’s expected revenue under various information-sharing strategies is compared based on the subgame in Proposition 7. From this comparison, the retailer’s optimal information-sharing strategies and subgame perfect equilibrium are derived.

**Proposition 7:** The retailer’s information-sharing strategies and the subgame perfect equilibrium are as follows:

(1) When $0<\beta <\beta_{1}$ and $\lambda_{2}\leq \lambda <\lambda_{3}$, the retailer chooses to share information, i.e., $E\left(\pi_{r}^{SA}\right)\geq E\left(\pi_{r}^{NA}\right)$. The subgame perfect equilibrium is (S,A). When $0<\beta <\beta_{1}$ and $0\leq \lambda <\lambda_{2}$, the retailer chooses not to share information, i.e., $E\left(\pi_{r}^{SA}\right)<E\left(\pi_{r}^{NA}\right)$. The subgame perfect equilibrium is (N,A). When $0<\beta <\beta_{1}$ and $\lambda_{3}\leq \lambda <1$, the retailer chooses not to share information, i.e., $E\left(\pi_{r}^{SR}\right)<E\left(\pi_{r}^{NR}\right)$. The subgame perfect equilibrium is (N,R).
(2) When $\beta_{1}\leq \beta <2/3$ and $0\leq \lambda <\lambda_{3}$, the retailer chooses not to share information, i.e., $E\left(\pi_{r}^{SA}\right)<E\left(\pi_{r}^{NA}\right)$. The subgame perfect equilibrium is (N,A). When $\beta_{1}\leq \beta <2/3$ and $\lambda_{3}\leq \lambda <1$, the retailer chooses not to share information, i.e., $E\left(\pi_{r}^{SR}\right)<E\left(\pi_{r}^{NR}\right)$. The subgame perfect equilibrium is (N,R).
(3) When $2/3\leq \beta <\beta_{3}$ and $0<\lambda <\lambda_{3}$, the retailer chooses not to share information, i.e., $E\left(\pi_{r}^{SA}\right)<E\left(\pi_{r}^{NA}\right)$. The subgame perfect equilibrium is (N,A). When (i) $2/3\leq \beta <\sqrt{3}-1$ and $\lambda_{3}\leq \lambda <1$, or (ii) $\sqrt{3}-1\leq \beta <\beta_{3}$ and $\lambda_{3}\leq \lambda <\lambda_{1}$ the retailer chooses not to share information, i.e., $E\left(\pi_{r}^{NR}\right)\geq E\left(\pi_{r}^{NA}\right)$. The subgame perfect equilibrium is (N,R).
(4) When $\beta_{3}\leq \beta <1$ and $0<\lambda <\lambda_{1}$, the retailer chooses not to share information, i.e., $E\left(\pi_{r}^{NR}\right)<E\left(\pi_{r}^{NA}\right)$. The subgame perfect equilibrium is (N,A).

According to Proposition 7, when the retailer’s information forecast accuracy is low or the market demand variance is small (i.e., $0<t\leq \min\left\{t_{1},1\right\}$), there is no significant first-mover advantage for either the manufacturer or the retailer. When the market demand variance is large, it indicates greater uncertainty in the market. However, because of the low information forecast accuracy, any first-mover advantage might be diminished due to prediction errors, making the advantage less evident. A smaller demand variance implies that consumer demand changes are minimal, which to some extent reduces the sensitivity of decision-makers in the market. Both parties find it difficult to gain an advantage from the expected benefits of moving first. However, when the market demand variance is large and the retailer’s information forecast accuracy is high (i.e., $t_{1}<t<1$), the first-mover advantage is highly apparent for the manufacturer. Within a certain threshold, the manufacturer can shift from a reselling mode to an agency selling mode by choosing to move first, prompting the retailer to share demand information more extensively, thereby profiting.

**Fig 4:**
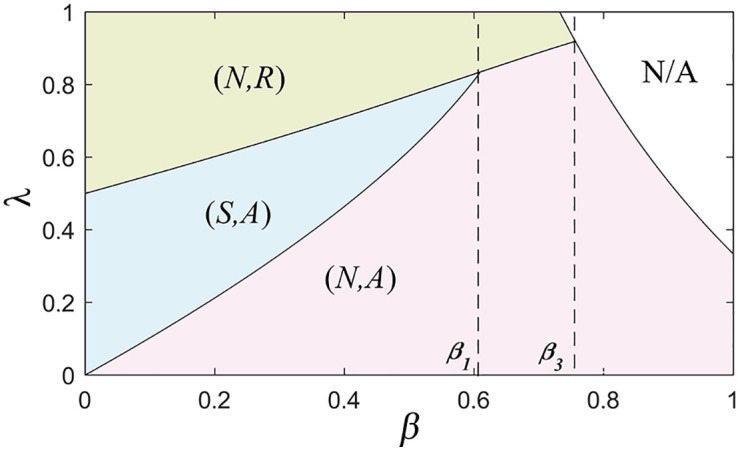
The subgame perfect equilibrium strategy.

The results of Proposition 7 are presented in Fig 4, showing how the commission rate and the product substitution rate affect equilibrium strategies. By contrasting Fig 4 with Fig 2(a), this comparison reflects that there is no significant first-mover advantage for either the manufacturer or the retailer when the retailer’s information forecast accuracy is low or the market demand variance is small (i.e., $0<t\leq \min\left\{t_{1},1\right\}$). However, when comparing Fig 4 with Fig 2(b), when the market demand variance is large and the retailer’s information forecast accuracy is high (i.e., $t_{1}<t<1$), the first-mover advantage is highly apparent for the manufacturer.

### 5.2. Market share difference

The basic model considers cases where the quality of the manufacturer’s brand and the retailer’s private label are equivalent. However, in real-world sales scenarios, retailers may choose to profit by introducing high-quality private labels [30]. For example, the Canadian retailer Loblaws has achieved a leading position in the Canadian market with its President’s Choice brand, known for its high-quality products, even surpassing the market share of some manufacturer brands [67]. This section will consider a situation where the retailer engages in a premium private label encroachment, i.e., $a_{m}<a_{r}$. To capture the asymmetry in market demand between the retailer’s private label and the manufacturer’s brand, θ (θ∈(0,1)) is used as the ratio of manufacturer brand market demand to retailer private label market demand [57], indicating that the retailer, due to its high-quality competitive advantage, has a higher market share.

For mathematical tractability, the market demand for the retailer’s private label is standardized as a ($a_{r}=a$), and the market demand for the manufacturer’s brand as θa ($a_{m}=\theta a$). Additionally, to ensure that the manufacturer’s brand and the retailer’s private label products coexist in the market, it is assumed that when $\sqrt{6}/3<\theta <1$ and $\sqrt{\theta^{2}+2}-\theta <\beta <\theta$, then $0<\lambda <\min\left\{\lambda_{8},1\right\}$, where $\lambda_{8}=\frac{4-\beta^{2}-2\theta \beta }{\beta \left(\beta +2\theta \right)}$. Otherwise, only the retailer’s private label products would exist in the reselling mode, or only the manufacturer’s branded products would be exclusively available under the agency selling mode. Other assumptions are the same as in the basic model.


$$p_{r}=a-q_{r}-\beta q_{m}$$ (21)

$$p_{m}=\theta a-q_{m}-\beta q_{r}$$ (22)

For clarity and conciseness in illustrating the subgame equilibrium results, two thresholds are defined:


$$\beta_{6}=2\theta /3$$

$$\lambda_{9}=\frac{3\beta^{3}-2\theta \;\beta^{2}-2\sqrt{4\beta^{4}\theta^{2}+3\beta^{4}-4\theta \;\beta^{3}-4\beta^{2}\theta^{2}+\beta^{2}-4\beta \theta +4\theta^{2}}-2\beta +4\theta }{3\beta^{2}\left(2\theta -\beta \right)}$$

The following proposition is derived.

**Proposition 8:** When $0<\beta <\beta_{6}$ and $\lambda_{9}<\lambda <1$, the retailer chooses to share market demand forecast information with the manufacturer in the agency selling mode, i.e., $E\left(\pi_{r}^{AS}\right)>E\left(\pi_{r}^{AN}\right)$; however, in the reselling mode, the retailer chooses not to share market demand forecast information, i.e., $E\left(\pi_{r}^{RS}\right)<E\left(\pi_{r}^{RN}\right)$.

Proposition 8 indicates that, when the retailer’s market share exceeds that of the manufacturer and the manufacturer selects the agency selling mode, there is still a certain range in which the retailer shares demand forecast information. Additionally, the threshold for the agency commission rate $\lambda_{9}$ increases with β (i.e., $\partial \lambda_{9}/\partial \beta >0$), a conclusion that remains consistent with the basic model. This indicates that as the product substitution rate increases, the retailer needs a higher commission rate to profit from the information-sharing agency selling mode. In the reselling mode, however, the retailer never benefits from sharing information.

For clarity and conciseness in presenting the final equilibrium results, six thresholds are defined:


$$\lambda_{10}=\frac{2\beta^{3}\theta -3\beta^{2}\theta^{2}-\beta^{2}+2\theta^{2}}{-2\beta^{4}+6\beta^{3}\theta -5\beta^{2}\theta^{2}+\beta^{2}-4\beta \theta +4\theta^{2}}$$

$$\lambda_{11}=\frac{-\beta^{4}+6\theta \;\beta^{3}-7\beta^{2}\theta^{2}-4\beta \theta +6\theta^{2}}{-3\beta^{4}+10\theta \;\beta^{3}-\left(9\theta^{2}-2\right)\beta^{2}-8\beta \theta +8\theta^{2}}$$

$$t_{5}=\frac{{a_{0}}^{2}\left(\left(-2\beta^{4}+6\theta \;\beta^{3}-5\beta^{2}\theta^{2}+\beta^{2}-4\beta \theta +4\theta^{2}\right)\lambda -2\theta \;\beta^{3}+3\beta^{2}\theta^{2}+\beta^{2}-2\theta^{2}\right)}{\sigma^{2}{\left(\beta -2\theta \right)}^{2}\left(\lambda -1\right)\left(\beta -1\right)\left(\beta +1\right)}$$

$\beta_{7}\in \left(0,\beta_{6}\right)$ is the solution of $\lambda_{10}-\lambda_{9}=0$.

$\beta_{8}\in \left(0,\beta_{6}\right)$ is the solution of $\lambda_{11}-\lambda_{9}=0$.

$\beta_{9}\in \left(\sqrt{\theta^{2}+2}-\theta ,\theta \right)$ is the solution of $\lambda_{10}-\lambda_{8}=0$.

**Proposition 9:** The manufacturer’s sales mode and the subgame perfect equilibrium are as follows:

(1) When $0<\beta <\beta_{7}$ and $0<\lambda <\lambda_{9}$, the manufacturer selects the agency selling mode, i.e., $E\left(\pi_{m}^{RN}\right)<E\left(\pi_{m}^{AN}\right)$. The subgame perfect equilibrium is (A,N); When $0<\beta <\beta_{7}$ and $\lambda_{9}\leq \lambda <\lambda_{10}$, the manufacturer selects the agency selling mode, i.e., $E\left(\pi_{m}^{RN}\right)<E\left(\pi_{m}^{AS}\right)$. The subgame perfect equilibrium is (A,S); When $0<\beta <\beta_{7}$, $\lambda_{10}\leq \lambda <\lambda_{11}$, and $0<t<\min\left\{t_{5},1\right\}$, the manufacturer selects the reselling mode, i.e., $E\left(\pi_{m}^{AS}\right)<E\left(\pi_{m}^{RN}\right)$. The subgame perfect equilibrium is (R,N); When $0<\beta <\beta_{7}$, $\lambda_{10}\leq \lambda <\lambda_{11}$ and $t_{5}<t<1$, the manufacturer selects the agency selling mode, i.e., $E\left(\pi_{m}^{RN}\right)<E\left(\pi_{m}^{AS}\right)$. The subgame perfect equilibrium is (A,S); When $0<\beta <\beta_{7}$ and $\lambda_{11}\leq \lambda <1$, the manufacturer selects the reselling mode, i.e., $E\left(\pi_{m}^{AS}\right)<E\left(\pi_{m}^{RN}\right)$. The subgame perfect equilibrium is (R,N).
(2) When $\beta_{7}\leq \beta <\beta_{8}$ and $0<\lambda <\lambda_{10}$, the manufacturer selects the agency selling mode, i.e., $E\left(\pi_{m}^{RN}\right)<E\left(\pi_{m}^{AN}\right)$. The subgame perfect equilibrium is (A,N); When $\beta_{7}\leq \beta <\beta_{8}$ and $\lambda_{10}<\lambda <\lambda_{9}$, the manufacturer selects the reselling mode, i.e., $E\left(\pi_{m}^{AN}\right)<E\left(\pi_{m}^{RN}\right)$. The subgame perfect equilibrium is (R,N); When $\beta_{7}\leq \beta <\beta_{8}$, $\lambda_{9}\leq \lambda <\lambda_{11}$ and $0<t<\min\left\{t_{5},1\right\}$, the manufacturer selects the reselling mode, i.e., $E\left(\pi_{m}^{AS}\right)<E\left(\pi_{m}^{RN}\right)$. The subgame perfect equilibrium is (R,N); When $\beta_{7}\leq \beta <\beta_{8}$, $\lambda_{9}\leq \lambda <\lambda_{11}$, and $t_{5}<t<1$, the manufacturer selects the agency selling mode, i.e., $E\left(\pi_{m}^{RN}\right)<E\left(\pi_{m}^{AS}\right)$. The subgame perfect equilibrium is (A,S). When $\beta_{7}\leq \beta <\beta_{8}$ and $\lambda_{11}\leq \lambda <1$, the manufacturer selects the reselling mode, i.e., $E\left(\pi_{m}^{AS}\right)<E\left(\pi_{m}^{RN}\right)$. The subgame perfect equilibrium is (R,N).
(3) When $\beta_{8}\leq \beta <\beta_{9}$ and $0\leq \lambda <\lambda_{10}$, the manufacturer selects the agency selling mode, i.e., $E\left(\pi_{m}^{RN}\right)<E\left(\pi_{m}^{AN}\right)$. The subgame perfect equilibrium is (A,N); When $\beta_{8}\leq \beta \leq \sqrt{\theta^{2}+2}-\theta$ and $\lambda_{10}\leq \lambda <1$; or $\sqrt{\theta^{2}+2}-\theta <\beta <\beta_{9}$ and $\lambda_{10}\leq \lambda <\lambda_{8}$, the manufacturer selects the reselling mode, i.e., $E\left(\pi_{m}^{AN}\right)\leq E\left(\pi_{m}^{RN}\right)$. The subgame perfect equilibrium is (R,N).
(4) When $\beta_{9}\leq \beta <\theta$ and $0\leq \lambda <\lambda_{8}$, the manufacturer selects the agency selling mode, i.e., $E\left(\pi_{m}^{RN}\right)<E\left(\pi_{m}^{AN}\right)$. The subgame perfect equilibrium is (A,N).

Proposition 9 indicates that, similar to the analysis in the basic model, the demand variance and the retailer’s information forecast accuracy influence the subgame perfect equilibrium. Additionally, when the retailer’s private label market share exceeds that of the manufacturer’s brand, the information forecast accuracy threshold that prompts a change in the manufacturer’s sales mode is lower than that when both parties have equal market shares, that is, $t_{5}<t_{1}$. This means that the retailer’s information forecast accuracy threshold is less than that in a scenario where the two products have equal market shares. However, the manufacturer still wants to obtain information through the agency selling mode within a certain threshold to enhance its competitiveness, thereby achieving higher revenues compared to the reselling mode.

**Fig 5:**
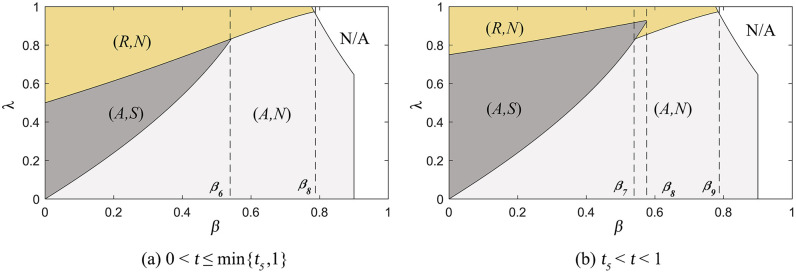
The subgame perfect equilibrium (θ=0.9).

The results of Proposition 9 are illustrated in Fig 5, demonstrating how the commission rate, the product substitution rate, and the retailer’s information forecasting accuracy impact equilibrium strategies under different market shares. Fig 5(a) depicts the subgame perfect equilibrium under scenarios where the retailer’s information forecast accuracy is low or the market demand variance is small (i.e., $0<t\leq \min\left\{t_{5},1\right\}$). Fig 5(b) depicts the subgame perfect equilibrium under scenarios where the market demand variance is larger and the retailer’s information forecast accuracy is high (i.e., $t_{5}<t<1$). The area in Fig 5(a) where the manufacturer selects the agency selling mode is larger than that in Fig 5(b). Specifically, when the product substitution rate is low, demand variance is high, and the commission rate is moderate, the manufacturer tends to shift from the reselling mode to the agency selling mode as the retailer’s information forecast accuracy improves. This conclusion is similar to that in Fig 2 (i.e., θ=1), and demonstrates the robustness of the basic model.

## 6. Numerical experiments

This section illustrates the impact of each parameter on the equilibrium strategies of both parties and the value of sharing information through numerical experiments. Without loss of generality, the constant part of the market size $a_{0}=1$ is assigned.

### 6.1. Information forecast accuracy in the reselling mode

**Fig 6:**
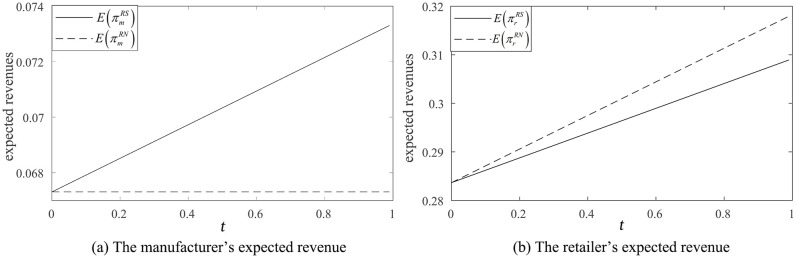
Impact of information forecast accuracy in the reselling mode ($\beta =0.3,\sigma^{2}$=0.09).

When the retailer chooses not to share market demand information in the reselling mode, the manufacturer can only set the wholesale price based on the fixed part of the market $a_{0}$ (Fig 6(a), dashed line). Therefore, the retailer’s information forecast accuracy does not impact the manufacturer’s revenue. However, when the retailer shares demand information, the manufacturer can make more informed decisions on the market wholesale price (Fig 6(a), solid line).

Fig 6(b) illustrates that the retailer’s expected revenue consistently increases as its information forecast accuracy improves. Higher information forecast accuracy enables the retailer to more effectively determine the quantities of the manufacturer’s products to resell and its private label products to sell, leading to higher expected revenue.

Furthermore, the retailer’s expected revenue with information-sharing (Fig 6(b) solid line) is always lower than when information is not shared (Fig 6(b) dashed line). Consequently, the retailer lacks any motivation to share its private forecast information.

### 6.2. Information forecast accuracy in the agency selling mode

**Fig 7:**
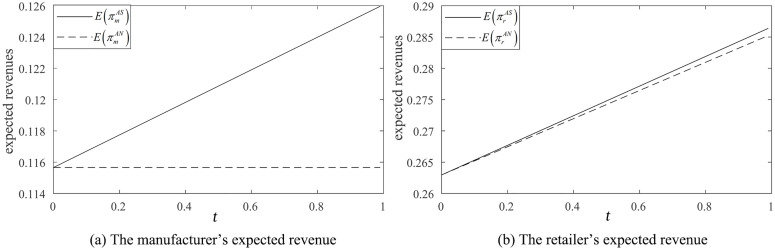
Impact of information forecast accuracy in agency selling (β=0.3, $\sigma^{2}=0.09,$ λ=0.4).

In the agency selling mode, the manufacturer benefits from the retailer sharing information, as illustrated in Fig 7(a) (the solid line is consistently higher than the dashed line). The information forecast accuracy is positively correlated with its benefit to both the manufacturer (Fig 7(a)) and retailer (Fig 7(b)).

When λ=0.4 and β=0.3, that is $\lambda >\lambda_{2}$, the retailer’s expected revenue with information-sharing (Fig 7(b) solid line) exceeds the revenue without information-sharing (Fig 7(b) dashed line), the retailer is incentivized to share the information with the manufacturer.

### 6.3. Commission rate in the agency selling mode

In the agency selling mode, the retailer’s information-sharing strategies depend on the product substitution rate β and the commission rate λ.

**Fig 8:**
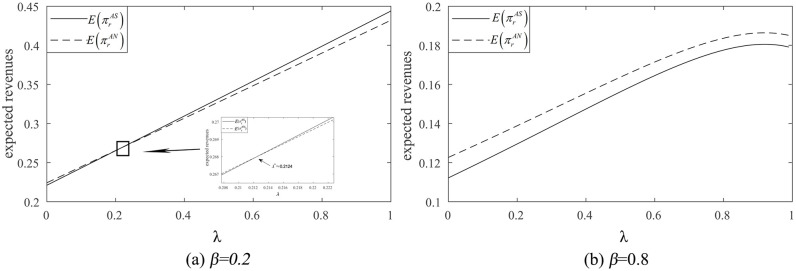
The impact of the commission rate on the retailer’s information-sharing strategies ($t=0.8,\sigma^{2}=0.09$).

In Fig 8(a), the retailer’s expected revenue increases with λ. When the product substitution rate β=0.2<2/3, a threshold for the commission rate $\lambda_{2}$ exists. If $\lambda <\lambda_{2}$, the retailer’s expected revenue from sharing information is less than that from not sharing. Conversely, if $\lambda_{2}\leq \lambda <1$, the retailer’s expected revenue from sharing information exceeds that of not sharing.

In Fig 8(b), when the product substitution rate β=0.8>2/3, the retailer’s expected revenue from not sharing is always larger than that from sharing. In this scenario, the retailer lacks any motivation to share its private forecast information.

### 6.4. Revenue-sharing rate and information forecast accuracy in the reselling mode

**Fig 9:**
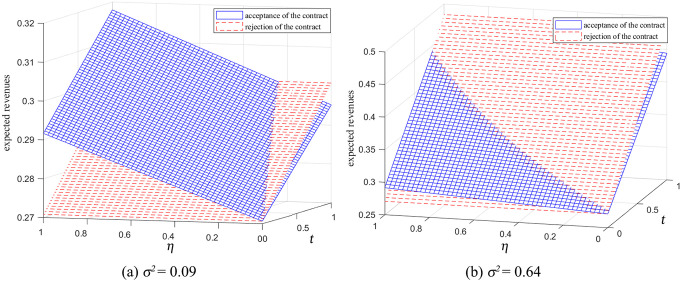
Impact of revenue-sharing rate and information forecast accuracy on the ICM in the reselling mode (β=0.5).

In the reselling mode, as the revenue-sharing rate η decreases, the retailer’s expected revenue from the ICM also decreases (Fig 9). Additionally, as the retailer’s information forecast accuracy t increases, the expected revenue from the ICM declines. In Fig 9(a), when $\sigma^{2}=0.09$ (i.e., $\sigma^{2}\leq a_{0}^{2}/2$), a revenue-sharing rate threshold $\eta_{1}$ exists. If $\eta_{1}<\eta <1$, the retailer’s expected revenue from accepting the mechanisms is greater than that from rejecting them. In Fig 9(b), when $\sigma^{2}=0.64$ (i.e., $a_{0}^{2}/2<\sigma^{2}<a_{0}^{2}$), the thresholds for the retailer’s information forecast accuracy and commission rate, $t_{3}$ and $\eta_{1}$ respectively, are established. If $0<t<t_{3}$ and $\eta_{1}<\eta <1$, the retailer will accept the manufacturer’s mechanisms. Moreover, Figs 9(a) and 9(b) also indicate that lower market demand variance or lower information forecast accuracy favors the cooperation of mechanisms.

### 6.5. Revenue-sharing rate and information forecast accuracy in the agency selling mode

**Fig 10:**
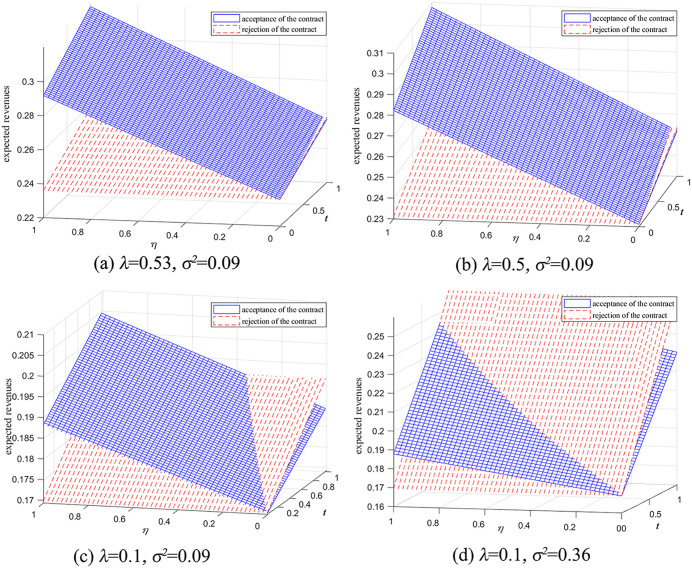
Impact of revenue-sharing rate and information forecast accuracy on the ICM in the agency selling mode (β=0.5).

In the agency selling mode (Figs 10(a) and 10(b)), when λ=0.53 (i.e., $\lambda_{6}\leq \lambda <\lambda_{2}$) and λ=0.5 (i.e., $\lambda_{7}\leq \lambda <\lambda_{6}$), a revenue-sharing rate threshold $\eta_{2}$ exists. If $\eta_{2}<\eta <1$, the retailer’s expected revenue from accepting the ICM is greater than that from rejecting it. Comparing Figs 10(c) and 10(d), when λ=0.1 (i.e., $0<\lambda <\lambda_{7}$), the interval within which the retailer accepts the ICM depends on the demand variance. When $\sigma^{2}=0.09$ (i.e., $t_{4}\approx 3.03>1$, Fig 10(c)), if $\eta_{2}<\eta <1$, the retailer’s expected revenue from the ICM exceeds that from rejection. Conversely, when $\sigma^{2}=0.36$ (i.e., $t_{4}<1$, Fig 10(d)), a threshold for the retailer’s information forecast accuracy $t_{4}$ exists, and the retailer will accept the manufacturer’s revenue-sharing ICM only if $0<t<t_{4}$ and $\eta_{2}<\eta <1$.

## 7. Conclusions

This paper examines the interaction between a manufacturer’s selection of selling mode and a retailer’s demand information-sharing strategy, with both parties competing in the same marketplace. The results illustrate the impact of factors such as commission rates, product competition intensity, demand variance, and information forecast accuracy on the choices made by both the retailer and the manufacturer. This research extends existing studies by relaxing the original assumptions that the demand for the manufacturer’s brands is always greater than that for the retailer’s brands and that the retailer has stronger channel power. This revised perspective offers new insights with substantial practical implications.

(1) Under the reselling mode, the retailer never chooses to share information with the manufacturer. In contrast, under the agency selling mode, the retailer chooses to share information when the commission rate is high and the product substitution rate is low. In the reselling mode, the sharing of information exacerbates the double marginalization issue, as the manufacturer can set the wholesale price in response to the demand signal. However, in the agency selling mode, sharing information enables the manufacturer to optimize the adjustment of sales quantities more effectively. When the commission rate is high and the product substitution rate is low, this adjustment outweighs the double marginalization effect, enabling the retailer to gain higher revenue from sharing information. Consequently, the retailer chooses to share information with the manufacturer under these conditions.
(2) Beyond the commission rate, the retailer’s information forecast accuracy, product substitution rate, and demand variance can influence the manufacturer’s selection of selling modes. Notably, when the product substitution rate is low, demand variance is high, and the commission rate is moderate, the manufacturer tends to shift from the reselling mode to the agency selling mode as the retailer’s information forecast accuracy improves. Additionally, when the commission rate is high, the manufacturer selects the reselling mode regardless of the product substitution rate, the retailer’s information forecast accuracy, or demand variance. This scenario is realistic, as the revenue gained from information would be less than the loss from costs if the manufacturer selects the agency selling mode under excessively high commission rates.
(3) The manufacturer consistently benefits from demand information-sharing in both reselling and agency selling modes. In a particular situation, the manufacturer selects the agency selling mode that can achieve a win-win outcome for both parties. This provides great insights for the manufacturer and the retailer. When demand variance is low, the win-win area for the manufacturer and the retailer is no less than when it is high at a certain level of information forecasting accuracy. Besides, the information compensation mechanism enables both the manufacturer and the retailer to reach a Pareto improvement.
(4) For the manufacturer and the retailer, when the retailer’s information forecast accuracy is low or market demand variability is small, the first-mover advantage is diminished. Therefore, whether the manufacturer or the retailer is the first to strategize, the equilibrium revenues of both parties remain unaffected. However, when the retailer’s information forecast accuracy is high and market demand variance is large, the first-mover advantage is apparent for the manufacturer within a certain threshold. Additionally, when the retailer’s private label market share exceeds that of the manufacturer’s brand, the threshold for the information forecast accuracy that prompts a change in the manufacturer’s sales mode is lower than the threshold for when both parties have equal market shares. Therefore, even when the retailer’s information forecasting accuracy is lower than the level where both parties have equal market shares, the manufacturer can still select the agency selling mode within a certain threshold range and benefit from it.

Manufacturers should avoid a one-size-fits-all strategy and instead tailor their approaches based on the retailers’ actions, product substitution rate, commission rate, demand variance, and retailers’ information forecast accuracy. Furthermore, selecting the agency selling mode can encourage retailers to share information, thus highlighting its positive effects.

This study has limitations and proposes possibilities for future studies. First, this paper assumes that private label products already exist in the market. However, it has not conducted in-depth analyses on the interaction mechanism among the manufacturer’s choices of selling modes, the retailer’s decisions on introducing private labels, and the retailer’s demand information sharing strategies under information asymmetry. Furthermore, in real-world scenarios, manufacturers may establish direct sales channels to enhance the market competitiveness of their products and compete with existing retail channels, and such scenarios also deserve exploration in the future.

## Supporting information

S1 File Proofs of Propositions. (DOCX)
