# Supplementary material for: Reselling or agency selling? Sales mode selection for a manufacturer in private label competition under information asymmetry
Source: PLoS One. 2026 Feb 3;21(2):e0341467. doi: 10.1371/journal.pone.0341467 (PMC12867271; doi:10.1371/journal.pone.0341467)
Supplement: S1 File — (DOCX) [file pone.0341467.s001.docx]

# **S1 Appendix. Proofs of Propositions**

**Proof for Proposition 1:**

In the reselling mode, let $\Delta_{1}=E\left(\pi_{r}^{RS}\right)-E\left(\pi_{r}^{RN}\right)=\frac{3\left(\beta-1\right)t\sigma^{2}}{16\beta+16}$. Given that $\beta\in\left(0,1\right)$, it follows that $\Delta_{1}=E\left(\pi_{r}^{RS}\right)-E\left(\pi_{r}^{RN}\right)<0$.

**Proof for Proposition 2:**

In the agency selling mode, if , there exists a feasible solution $0<\lambda<\min\left\{\lambda_{1}\text{，}1\right\}$, such that both store brand and manufacturer brand co-exist in the market, where $\lambda_{1}=\frac{-\beta^{2}-2\beta+4}{\beta^{2}+2\beta}$.

Let $\Delta_{2}=E\left(\pi_{r}^{AS}\right)-E\left(\pi_{r}^{AN}\right)$, and we obtain: $\Delta_{2}=$ $\frac{h_{1}t\sigma^{2}\left(2-\beta\right)}{16\left(-2+\beta^{2}\left(\lambda+1\right)\right)^{2}}$, where $h_{1}=3\left(\lambda+1\right)^{2}\beta^{3}+\left(-6\lambda^{2}-4\lambda+2\right)\beta^{2}+\left(-4\lambda-8\right)\beta+8\lambda$. Given that $\beta\in\left(0,1\right)$ and $16\left(-2+\beta^{2}\left(\lambda+1\right)\right)^{2}>0$, the positivity or negativity of $h_{1}$ must be analyzed. Let yielding $\frac{\partial^{2}h_{1}}{\partial\lambda^{2}}=6\beta^{2}\left(\beta-2\right)<0$, so $h_{1}$ is a concave function.

When $\lambda\in\left(\frac{3\beta^{3}-2\beta^{2}-2F_{1}-2\beta+4}{3\beta^{2}\left(2-\beta\right)},\right.\left.\frac{3\beta^{3}-2\beta^{2}+2F_{1}-2\beta+4}{3\beta^{2}\left(2-\beta\right)}\right)$, it follows that $E\left(\pi_{r}^{AS}\right)-E\left(\pi_{r}^{AN}\right)>0$, where $F_{1}=\sqrt{7\beta^{4}-4\beta^{3}-3\beta^{2}-4\beta+4}$. Additionally, it is proven that $\frac{3\beta^{3}-2\beta^{2}+2F_{1}-2\beta+4}{3\beta^{2}\left(2-\beta\right)}\notin\left(0,1\right)$. It is necessary to determine whether $\frac{3\beta^{3}-2\beta^{2}-2F_{1}-2\beta+4}{3\beta^{2}\left(2-\beta\right)}\in\left(0,1\right)$.When$\beta\to0^{+}$, then $3\beta^{2}\left(2-\beta\right)>0$. Because of $\left(3\beta^{3}-2\beta^{2}-2\beta+4\right)^{2}-\left(2F_{1}\right)^{2}=9\beta^{6}-12\beta^{5}-36\beta^{4}+$ $48\beta^{3}>0$, we can get $\frac{3\beta^{3}-2\beta^{2}-2F_{1}-2\beta+4}{3\beta^{2}\left(2-\beta\right)}>0$. Let , resulting in the first-order $\frac{\partial\lambda_{2}}{\partial\beta}=\frac{\left(4\beta^{3}-4\beta^{2}+16\beta-16\right)F_{1}-14\beta^{5}+12\beta^{4}+4\beta^{3}8\beta^{2}-48\beta+32}{3F_{1} \beta^{3}\left(\beta-2\right)^{2}}$. When $\beta\in$ $\left(0,\frac{\left(97+56\sqrt{3}\right)^{\frac{2}{3}}-\left(97+56\sqrt{3}\right)^{\frac{1}{3}}+1}{7\left(97+56\sqrt{3}\right)^{\frac{1}{3}}}\right)$, resulting in $\frac{\partial\lambda_{2}}{\partial\beta}>0$, $\lambda_{2}$ is increasing as $\beta$ increases. Solving $\frac{3\beta^{3}-2\beta^{2}-2F_{1}-2\beta+4}{3\beta^{2}\left(2-\beta\right)}=1$, resulting in $\beta=\frac{2}{3}$. As a result, when $\beta<\frac{2}{3}$ and $\lambda_{2}<\lambda<1$ ($\frac{\left(97+56\sqrt{3}\right)^{\frac{2}{3}}-\left(97+56\sqrt{3}\right)^{\frac{1}{3}}+1}{7\left(97+56\sqrt{3}\right)^{\frac{1}{3}}}>\frac{2}{3}$), it follows that $E\left(\pi_{r}^{AS}\right)>E\left(\pi_{r}^{AN}\right)$, and the retailer will share market demand forecast information with the manufacturer under the agency selling mode.

**Proof for Proposition 3:**

Based on Proposition 1 and Proposition 2, when the manufacturer chooses the reselling mode, the retailer does not share information with the manufacturer. However, in the agency selling mode, if $0<\beta<\frac{2}{3}$ and $\lambda_{2}<\lambda<1$, the retailer will share information. When $\frac{2}{3}\leq\beta<1$ or $0<\beta<\frac{2}{3}$ and $0<\lambda<\lambda_{2}$, whether the manufacturer chooses the reselling or agency selling mode, the retailer will not share market demand information.

When $0<\beta<\frac{2}{3}$ and $\lambda_{2}<\lambda<1$, let $\Delta_{3}=E\left(\pi_{m}^{AS}\right)-E\left(\pi_{m}^{RN}\right)$, and we obtain $\Delta_{3}$ $=\frac{\left(1-\lambda\right)\left(a_{0}^{2}+t\sigma^{2}\right)\left(\beta-2\right)^{2}}{16-\left(8\lambda+8\right)\beta^{2}}-\frac{a_{0}^{2}\left(1-\beta\right)}{8\beta+8}$. It follows that $\frac{\partial\Delta_{3}}{\partial t}=\frac{\left(1-\lambda\right)\sigma^{2}\left(\beta-2\right)^{2}}{16-\left(8\lambda+8\right)\beta^{2}}$ $>0$. Solving $\Delta_{3}=0$, we obtain $t=\frac{2a_{0}^{2}h_{2}}{\left(\beta+1\right)\left(1-\lambda\right)\sigma^{2}\left(\beta-2\right)^{2}}$, where $h_{2}=\lambda\beta^{3}+$ $\left(1-2\lambda\right)\beta^{2}-\beta+2\lambda-1$. When $t\geq\frac{2a_{0}^{2}h_{2}}{\left(\beta+1\right)\left(1-\lambda\right)\sigma^{2}\left(\beta-2\right)^{2}}$, then $E\left(\pi_{m}^{AS}\right)\geq$ $E\left(\pi_{m}^{RN}\right)$. Setting $t_{1}=\frac{2a_{0}^{2}h_{2}}{\sigma^{2}\left(1+\beta\right)\cdot\left(1-\lambda\right)\cdot\left(2-\beta\right)^{2}}$, and given that $\lambda\in\left(0,1\right)$, the positivity or negativity of $h_{2}$ is needs to be discussed. Taking the partial derivative of $h_{2}$ with respect to $\lambda$, we obtain $\frac{\partial h_{2}}{\partial\lambda}=\beta^{3}-2\beta^{2}+2>0$. By solving $h_{2}=0$, we find when $\lambda>\lambda_{3}$, then $t_{1}>0$, where $\lambda_{3}=\frac{1+\beta-\beta^{2}}{\beta^{3}-2\beta^{2}+2}$. Furthermore, when $\lambda>\lambda_{3}$, the range of values for $\frac{2h_{2}}{\left(1+\beta\right)\left(1-\lambda\right)\left(2-\beta\right)^{2}}$ must be determined (because of $\frac{a_{0}^{2}}{\sigma^{2}}>$ $1$, if $\frac{2h_{2}}{\left(1+\beta\right)\left(1-\lambda\right)\left(2-\beta\right)^{2}}\geq1$, then $t_{1}>1$). Setting $h_{3}=2h_{2}-\left(1+\beta\right)\left(1-\lambda\right)\left(2-\beta\right)^{2}$, we find $\frac{\partial h_{3}}{\partial\lambda}=3\beta^{3}-7\beta^{2}+8>0$. Given $\lambda_{3}<\lambda<1$, substituting $\lambda=1$ into $h_{3}$ yields a maximum value for $h_{3}=2 \beta^{3}-2\beta^{2}-2\beta+2>0$, and substituting $\lambda=\lambda_{3}$ into $h_{3}$, and get the minimum $h_{3}\left(\lambda=\lambda_{3}\right)=\left(3\lambda-1\right)\beta^{3}+\left(-7\lambda+5\right)\beta^{2}-2\beta+8\lambda-6$. Given $\beta\in\left(0,\frac{2}{3}\right)$, resulting in $\frac{\partial h_{3}\left(\lambda_{3}\right)}{\partial\beta}>0$. Similarly, solving $h_{3}\left(\lambda_{4}\right)=0$, we find $\lambda_{4}=$ $\frac{\beta^{3}-5\beta^{2}+2\beta+6}{3\beta^{3}-7\beta^{2}+8}$. When $\lambda_{3}<\lambda<\lambda_{4}$, then $h_{3}<0$; when $\lambda_{4}\leq\lambda<1$, then $h_{3}\geq0$ (i.e., $t_{1}>1$). Besides, we need to compare $\lambda_{2}$, $\lambda_{3}$ and $\lambda_{4}$. As can be seen from the above, $\lambda_{4}>\lambda_{3}$ ($0<\beta<\frac{2}{3}$).

Setting $\Delta_{\lambda_{32}}=\lambda_{3}-\lambda_{2}$, and obtain $\Delta_{\lambda_{32}}=\frac{\left(-2\beta^{3}+4\beta^{2}-4\right)F_{1}+F_{2}}{3\beta^{2}\left(\beta-2\right)\left(\beta^{3}-2\beta^{2}+2\right)}$, where $F_{2}=3\beta^{6}-11\beta^{5}+11\beta^{4}+11\beta^{3}-18\beta^{2}-4\beta+8$. When $0<\beta<\frac{2}{3}$, then $\frac{\partial\Delta_{\lambda_{32}}}{\partial\beta}<0$. Substituting $\beta=\frac{2}{3}$ into $\Delta_{\lambda_{32}}$, we have the minimum $\Delta_{\lambda_{32}}=-\frac{5}{38}<0$, and substituting $\beta\to0^{+}$ into $\Delta_{\lambda_{32}}$, we obtain the maximum $\Delta_{\lambda_{32}}=\frac{1}{2}>0$. According to the zero theorem, there exists $\beta_{1}$, and have $\Delta_{\lambda_{32}}\left(\beta_{1}\right)=0$, where $\beta_{1}$ ($\beta_{1}\approx0.6099<\frac{2}{3}$) is a solution of $\Delta_{\lambda_{32}}=0$. When $\beta_{1}\leq\beta<\frac{2}{3}$, then $\lambda_{2}\geq\lambda_{3}$; when $0<\beta<\beta_{1}$, then $\lambda_{2}<\lambda_{3}$.

Setting $\Delta_{\lambda_{42}}=\lambda_{4}-\lambda_{2}$, $\Delta_{\lambda_{42}}=\frac{-4\left(1-\beta\right)^{2}\left(8+\beta\left(12+\beta\left(-4+3\left(-2+\beta\right)\beta\right)\right)\right)}{3\left(3\beta^{3}-7\beta^{2}+8\right)\beta^{2}\left(2-\beta\right)}+$ $\frac{2F_{1}}{3\beta^{2}\left(2-\beta\right)}$. Because of $\frac{\partial\Delta_{\lambda_{42}}}{\partial\beta}<0$, substituting $\beta\to0^{+}$ into $\Delta_{42}$ yields a maximum $\Delta_{42}=\frac{3}{4}$ , and substituting $\beta=\frac{2}{3}$ into $\Delta_{42}$, and get the minimum$\Delta_{42}=-\frac{5}{78}$. According to the zero theorem, there exists $\beta_{2}$, and have $\Delta_{42}\left(\beta_{2}\right)=0$, where $\beta_{2}$ ($\beta_{2}\approx\text{ 0.6456}$) is a solution of $\Delta_{42}=0$. When $0<\beta<\beta_{2}$, then $\lambda_{4}>\lambda_{2}$; when $\beta_{2}\leq\beta<\frac{2}{3}$, then $\lambda_{4}\leq\lambda_{2}$. When $\frac{2}{3}\leq\beta<1$, setting $\Delta_{4}=E\left(\pi_{m}^{RN}\right)-$ $E\left(\pi_{m}^{AN}\right)=$ $\frac{a_{0}^{2}\left(\lambda\beta^{3}+\left(1-2\lambda\right)\left(\beta^{2}-1\right)-\beta\right)}{4\left(\beta+1\right)\left(2-\left(1+\lambda\right)\beta^{2}\right)}$, we find . Substituting $\lambda=1$ into $\Delta_{4}$ yields a maximum value for $\Delta_{4}=\frac{a_{0}^{2}\left(\beta^{3}-\beta^{2}-\beta+1\right)}{4\left(\beta+1\right)\left(2-2\beta^{2}\right)}$ $>0$, and substituting $\lambda=0$ into $\Delta_{4}$, and get the minimum value for$\Delta_{4}=$ $\frac{a_{0}^{2}\left(\beta^{2}-\beta-1\right)}{4\left(\beta+1\right)\left(2-\beta^{2}\right)}<0$ . According to the zero theorem, when $\frac{2}{3}\leq\beta<1$, there exists $\lambda_{3}=\frac{1+\beta-\beta^{2}}{\beta^{3}-2\beta^{2}+2}$ that $\Delta_{4}\left(\lambda_{3}\right)=0$ . When $\lambda_{3}\leq\lambda<1$ , then $\Delta_{4}\geq0$ ; When $0<\lambda<\lambda_{3}$ , then $\Delta_{4}\leq0$ .When $\lambda_{3}\leq\lambda<1$,the manufacturer chooses the reselling mode and when $0<\lambda<\lambda_{3}$, the manufacturer will choose the agency selling mode. Because of $0<\lambda<\min\left\{\lambda_{1}\text{，}1\right\}$ (), by comparing $\lambda_{1}$ and $\lambda_{3}$, we have $\Delta_{\lambda_{13}}=\lambda_{1}-\lambda_{3}=$ $\frac{\beta^{5}-\beta^{4}-9\beta^{3}+13\beta^{2}+6\beta-8}{\left(\beta^{3}-2\beta^{2}+2\right)\beta\left(\beta+2\right)}$. When , then $\frac{\partial\Delta_{\lambda_{13}}}{\partial\beta}>0$. substituting $\beta=1$ into $\Delta_{\lambda_{13}}$ yields a maximum value for $\Delta_{\lambda_{13}}=\frac{2}{3}$, and substituting $\beta=\sqrt{3}-1$ into $\Delta_{\lambda_{13}}$, and get the minimum value for $\Delta_{\lambda_{13}}=\frac{-7\sqrt{3}+12}{10\sqrt{3}-16}<0$. According to the zero theorem, when , there exists $\beta_{3}$ ($\beta_{3}\approx0.756$) that $\Delta_{\lambda_{13}}\left(\beta_{3}\right)=0$. When , then $\lambda_{1}<\lambda_{3}$; When , then $\lambda_{1}\geq\lambda_{3}$. Similarly, when $0<\beta<\frac{2}{3}$ and $0<\lambda<\lambda_{2}$, because of $\frac{\partial\Delta_{4}}{\partial\lambda}>0$, substituting $\lambda=\lambda_{2}$ into $\Delta_{4}$ yields a maximum value for $\Delta_{4\max}=\frac{a_{0}^{2}\left(\left(2\beta^{3}-4\beta^{2}+4\right)F_{1}-F_{2}\right)}{8\left(2\beta^{2}+2\beta-F_{1}-4\right)\beta^{2}\left(\beta+1\right)}$. When $0<$ $\beta<\frac{2}{3}$, then $\frac{\partial\Delta_{4\max}}{\partial\beta}>0$. Substituting $\beta=\frac{2}{3}$ into $\Delta_{4\max}$, we have $\Delta_{4\max}=\frac{a_{0}^{2}}{40}>0$, and substituting $\beta\to0^{+}$ into $\Delta_{4\max}$, we obtain $\Delta_{4\max}=-\frac{1}{8}a_{0}^{2}<0$. According to the zero theorem, there exists $\beta_{1}$, when $\Delta_{4\max}\left(\beta_{1}\right)=0$, where $\beta_{1}$ ($\beta_{1}\approx0.6099<\frac{2}{3}$) is a solution of $\Delta_{_{4\max}}=0$. When $0<\beta$ $<\beta_{1}$, then $\Delta_{4}<0$; When $\beta_{1}\leq\beta<\frac{2}{3}$, there exists $\lambda_{3}$ that $\Delta_{4}\left(\lambda_{3}\right)$ $=0$. When $\lambda_{3}<\lambda<\lambda_{2}$, then $\Delta_{4}>0$; When $\lambda\leq\lambda_{3}$, then $\Delta_{4}\leq0$.

**Proof for Proposition 4:**

In the reselling mode, we obtain $E\left(\pi_{m}^{RS}\right)-E\left(\pi_{m}^{RN}\right)=\frac{\left(1-\beta\right)t\sigma^{2}}{8\beta+8}$,

Similarly, in the agency selling mode, we find:

$$E\left(\pi_{m}^{AS}\right)-E\left(\pi_{m}^{AN}\right)=\frac{\left(1-\lambda\right)t\sigma^{2}\left(\beta-2\right)^{2}}{16-\left(8\lambda+8\right)\beta^{2}}$$

Given the $0<\beta<1$ and $0<\lambda<1$, it can be deduced that $E\left(\pi_{m}^{RS}\right)>E\left(\pi_{m}^{RN}\right)$and $E\left(\pi_{m}^{AS}\right)>E\left(\pi_{m}^{AN}\right)$. Therefore, whether the manufacturer selects the reselling or agency selling mode, the retailer’s information sharing leads to an increase in the manufacturer's expected revenue.

**Proof for Proposition 5:**

In addition, in conjunction with the conclusion of Proposition 3 and Proposition 4, the win-win approach can be conducted in Proposition 5.

(1) We set $\Delta_{5}=E\left(\pi_{m}^{AS}\right)-E\left(\pi_{m}^{RN}\right)=\frac{\left(t\sigma^{2}+a_{0}^{2}\right)\left(\beta^{3}\lambda+\left(-2\lambda+1\right)\left(\beta^{2}-1\right)-\beta\right)}{4\left(\beta+1\right)\left(2-\left(\lambda+1\right)\beta^{2}\right)}$. Similar to Proposition 3, when $\lambda<\lambda_{3}$, then $E\left(\pi_{m}^{AS}\right)>E\left(\pi_{m}^{RN}\right)$; when $\lambda\geq\lambda_{3}$, then $E\left(\pi_{m}^{AS}\right)\leq E\left(\pi_{m}^{RN}\right)$.

(2) Because of $E\left(\pi_{r}^{RS}\right)<E\left(\pi_{r}^{RN}\right)$, we need to compare $E\left(\pi_{r}^{RN}\right)$ and $E\left(\pi_{r}^{AS}\right)$, set $\Delta_{6}=E\left(\pi_{r}^{AS}\right)-E\left(\pi_{r}^{RN}\right)$:

$$\Delta_{6}=-\frac{\left(\left(\lambda+1\right)^{2}\beta^{5}+\left(-4\lambda^{2}-4\lambda\right)\beta^{4}+\left(-4\lambda-6\right)\beta^{3}+\left(6\lambda^{2}+8\lambda\right)\beta^{2}+6\beta-8\lambda+2\right)a_{0}^{2}}{8\left(\beta+1\right)\left(-2+\left(\lambda+1\right)\beta^{2}\right)^{2}}$$

$$-\frac{\left(\left(\lambda+1\right)^{2}\beta^{5}+\left(-4\lambda^{2}-4\lambda\right)\beta^{4}+\left(-4\lambda-6\right)\beta^{3}+\left(6\lambda^{2}+8\lambda\right)\beta^{2}+6\beta-8\lambda+2\right)a_{0}^{2}}{8\left(\beta+1\right)\left(-2+\left(\lambda+1\right)\beta^{2}\right)^{2}}$$

Taking the partial derivative of $\Delta_{6}$ with respect to $t$, we get $\frac{\partial\Delta_{5}}{\partial t}=$ $\frac{\left(\left(\lambda+1\right)^{2}\beta^{5}+\left(5\lambda^{2}+2\lambda-3\right)\beta^{4}-4\beta^{3}\lambda+\left(-12\lambda^{2}-4\lambda+12\right)\beta^{2}+16\lambda-16\right)\sigma^{2}}{16\left(-2+\left(\lambda+1\right)\beta^{2}\right)^{2}\left(\beta+1\right)}$. Setting $g_{1}=\left(\lambda+1\right)^{2}\beta^{5}+\left(5\lambda^{2}+2\lambda-3\right)\beta^{4}-4\beta^{3}\lambda+\left(-12\lambda^{2}-4\lambda+12\right)\beta^{2}+16\lambda-16$, we have $\frac{\partial g_{1}}{\partial\lambda}=2\left(\beta+2\right)\left(\left(\lambda+1\right)\beta^{4}+\left(3\lambda-1\right)\beta^{3}-6\beta^{2}\lambda-2\beta+4\right)$. Taking the second-order partial derivative of $g_{1}$ with respect to $\lambda$, we get $\frac{\partial^{2}g_{1}}{\partial\lambda^{2}}=2\beta^{5}+10\beta^{4}-24\beta^{2}<0$. Substitute $\lambda_{3}$ to $\frac{\partial g_{1}}{\partial\lambda}$, we have $\frac{\partial g_{1}}{\partial\lambda}>\frac{2\left(2-\beta\right)\left(\beta+1\right)\left(2+\beta\right)\left(4+\beta\left(4+\left(1-\beta\right)\beta\right)\right)}{2+\left(\beta-2\right)\beta^{2}}$

$*\left(\beta-1\right)^{2}>0$. Substitute $\lambda_{3}$ to $g_{1}$, then $g_{1}<\frac{\beta^{6}-6\beta^{5}+4\beta^{4}+14\beta^{3}-9\beta^{2}-8\beta+8}{\left(\beta^{3}-2\beta^{2}+2\right)^{2}}$ $*\left(\beta+1\right)^{2}\left(\beta-2\right)^{2}\left(\beta-1\right)<0$, and obtain $\frac{\partial\Delta_{6}}{\partial t}<0$.

Substitute $t=0$ to $\Delta_{6}$, we can get $\Delta_{6}\left(t=0\right)=\frac{a_{0}^{2}g_{2}}{8\left(-2+\left(\lambda+1\right)\beta^{2}\right)^{2}\left(\beta+1\right)}$, where $g_{2}=-\left(\lambda+1\right)^{2}\beta^{5}+\left(4\lambda^{2}+4\lambda\right)\beta^{4}+\left(4\lambda+6\right)\beta^{3}-\left(6\lambda^{2}+8\lambda\right)\beta^{2}-6\beta+8\lambda-2$. Similarly, we have $\frac{\partial g_{2}}{\partial\lambda}=-2\left(\lambda+1\right)\beta^{5}-\left(-8\lambda-4\right)\beta^{4}+4\beta^{3}-\left(12\lambda+8\right)\beta^{2}+8$, and we can get $\frac{\partial^{2}g_{2}}{\partial\lambda^{2}}=-2\beta^{5}+8\beta^{4}-12\beta^{2}<0$. Substitute$\lambda_{3}$ to $\frac{\partial g_{2}}{\partial\lambda}$, we have $\frac{\partial g_{2}}{\partial\lambda}>$ $\frac{2\left(2-\beta\right)\left(\beta+1\right)\left(\beta^{4}-2\beta^{3}+6\beta+4\right)\left(\beta-1\right)^{2}}{\beta^{3}-2\beta^{2}+2}>0$. Substitute $\lambda_{3}$ to $g_{2}$, we can get $g_{2}\left(\lambda=\lambda_{3}\right)=\frac{\left(1-\beta\right)\left(\beta^{6}-3\beta^{5}+\beta^{4}+5\beta^{3}-6\beta^{2}-2\beta+2\right)\left(\beta+1\right)^{2}\left(2-\beta\right)^{2}}{\left(\beta^{3}-2\beta^{2}+2\right)^{2}}$. There exist $\beta_{4}$($\beta_{4}\approx0.524$), when $0<\beta<\beta_{4}$, then $g_{2}\left(\lambda=\lambda_{3}\right)>0$; when $\beta_{4}\leq\beta<\beta_{1}$, then $g_{2}\left(\lambda=\lambda_{3}\right)\leq0$. Substitute $\lambda_{2}$ to $g_{2}$, we can get $g_{2}\left(\lambda=\lambda_{2}\right)=\left(1-\beta\right)^{2}$ $*\frac{2\left(\left(-4\beta-8\right)\sqrt{7\beta^{4}-4\beta^{3}-3\beta^{2}-4\beta+4}+11\beta^{3}+15\beta^{2}-20\right)}{9\left(-2+\beta\right)^{2}}<0$. When $0<\beta<\beta_{4}$, there exist $\lambda_{5}=\frac{\left(\beta^{2}-\beta-2\right)\sqrt{6\beta^{4}-2\beta^{3}-4\beta^{2}-4\beta+4}-\beta^{5}+2\beta^{4}+2\beta^{3}-4\beta^{2}+4}{\beta^{2}\left(\beta^{3}-4\beta^{2}+6\right)}$, when $\lambda_{2}\leq\lambda\leq\lambda_{5}$, then $g_{2}\leq0$, when $\lambda_{5}<\lambda<\lambda_{3}$, then $g_{2}>0$ (which means $\Delta_{6}\left(t=0\right)>0$).

When $\Delta_{6}\left(t=0\right)\leq0$, then $\Delta_{6}<0$. When $\Delta_{6}\left(t=0\right)>0$, there exists the $t_{2}$, that if $0<t<\min\left\{1,t_{2}\right\}$, then $\Delta_{6}>0$; if $t\geq t_{2}$, then $\Delta_{6}\leq0$, where $t_{2}=$ $\frac{2\left(\left(\lambda+1\right)^{2}\beta^{5}+\left(-4\lambda^{2}-4\lambda\right)\beta^{4}+\left(-4\lambda-6\right)\beta^{3}+\left(6\lambda^{2}+8\lambda\right)\beta^{2}+6\beta-8\lambda+2\right)a_{0}^{2}}{\left(\left(\lambda+1\right)^{2}\beta^{5}+\left(5\lambda^{2}+2\lambda-3\right)\beta^{4}-4\beta^{3}\lambda+\left(-12\lambda^{2}-4\lambda+12\right)\beta^{2}+16\lambda-16\right)\sigma^{2}}$.

In conjunction with the conclusion of Proposition 3, Proposition 4, and Proposition 5, we can derive that when $0<\beta<\beta_{4}$, $\lambda_{5}<\lambda<\lambda_{3}$ and $0<t<\min\left\{t_{2},1\right\}$, a win-win approach for both retailer and manufacturer can be achieved under the agency selling mode.

**Proof for Proposition 6:**

A coordination mechanism is introduced for supply chains to determine the conditions necessary for information-compensated coordination. This mechanism employs revenue-sharing contracts to allocate the expected revenue increase of the entire supply chain, under centralized decision-making, to retailers and manufacturers in proportions $\eta$ and $1-\eta$, respectively. The total expected revenue of the supply chain under centralized decision-making is given by $E\left(\pi_{T}^{TC}\right)=\frac{a_{0}^{2}+t\sigma^{2}}{2\beta+2}$.

(1) In the reselling mode, when the retailer shares information, resulting in the total expected revenue of the supply chain is: $E\left(\pi_{T}^{RS}\right)=\frac{\left(a_{0}^{2}+t\sigma^{2}\right)\left(\beta+7\right)}{16\beta+16}$. Setting $\Delta_{7}=\Delta^{R}=$ $E\left(\pi_{T}^{TC}\right)-E\left(\pi_{T}^{RS}\right)$, we obtain $\Delta_{7}=E\left(\pi_{T}^{TC}\right)-E\left(\pi_{T}^{RS}\right)=\frac{\left(a_{0}^{2}+t\sigma^{2}\right)\left(1-\beta\right)}{16\beta+16}$ $>0$.

The manufacturer’s gain from the contract is $E\left(\pi_{m}^{RSC}\right)=\frac{\left(1-\beta\right)(1-\eta)\left(a_{0}^{2}+t\sigma^{2}\right)}{16\beta+16}$ $+\frac{\left(1-\beta\right)\left(a_{0}^{2}+t\sigma^{2}\right)}{8\beta+8}$. The retailer’s gain from the contract is $E\left(\pi_{r}^{RSC}\right)=$ $\frac{\left(t\sigma^{2}+a_{0}^{2}\right)\left(\left(3\beta+5\right)+\eta\left(1-\beta\right)\right)}{16\beta+16}$. For the contract to be feasible, the following condition must be satisfied: $E\left(\pi_{r}^{RSC}\right)>E\left(\pi_{r}^{RN}\right)$. Setting $\Delta_{8}=E\left(\pi_{r}^{RSC}\right)-E\left(\pi_{r}^{RN}\right)$, and have $\Delta_{8}=E\left(\pi_{r}^{RSC}\right)-E\left(\pi_{r}^{RN}\right)=\frac{\left(t\left(\eta-3\right)\sigma^{2}+a_{0}^{2}\eta\right)\left(1-\beta\right)}{16\beta+16}$. We find the first-order derivative of $\Delta_{8}$ with $\eta$, resulting in $\frac{\partial\Delta_{8}}{\partial\eta}>0$. Solving $\Delta_{8}=0$, we obtain $\eta=\frac{3t\sigma^{2}}{t\sigma^{2}+a_{0}^{2}}$. Setting $\eta_{1}=\frac{3t\sigma^{2}}{t\sigma^{2}+a_{0}^{2}}$, if $\eta\in\left(\eta_{1},1\right)$ and $\eta_{1}<1$, then $E\left(\pi_{r}^{RSC}\right)>$ $E\left(\pi_{r}^{RN}\right)$. When $t<\frac{a_{0}^{2}}{2\sigma^{2}}$, then $\eta_{1}<1$, and setting $t_{3}=\frac{a_{0}^{2}}{2\sigma^{2}}$.

(2) In the agency selling mode, when the retailer sharing information, the total expected revenue of the supply chain is given by $E\left(\pi_{T}^{AS}\right)=\frac{\left(a_{0}^{2}+t\sigma^{2}\right)F_{3}}{16\left(-2+\beta^{2}\left(\lambda+1\right)\right)^{2}}$, where $F_{3}=32+\left(3\lambda^{2}+2\lambda-1\right)\beta^{4}+\left(4\lambda^{2}+16\lambda+12\right)\beta^{3}+\left(-4\lambda^{2}-24\lambda-8\right)\beta^{2}-32\beta$. Setting $\Delta_{9}=\Delta^{A}=E\left(\pi_{T}^{TC}\right)-E\left(\pi_{T}^{AS}\right)$, we obtain $\Delta_{9}=\frac{\left(t\sigma^{2}+a_{0}^{2}\right)\beta^{2}F_{4}}{16\left(1+\beta\right)\left(-2+\beta^{2}\left(\lambda+1\right)\right)^{2}}$, where $F_{4}=-3\beta^{3}\lambda^{2}-2\beta^{3}\lambda+\beta^{2}\lambda^{2}+\beta^{3}-2\beta^{2}\lambda-3\beta^{2}+8\beta\lambda+4\lambda^{2}-4\beta-8\lambda+8$. Given the $\beta\in\left(0,1\right)$ and $\lambda\in\left(0,1\right)$, it follows that $\Delta_{9}>0$.

The retailer’s gain from the contract is: $E\left(\pi_{r}^{ASC}\right)=\frac{\eta\left(t\sigma^{2}+a_{0}^{2}\right)\beta^{2}F_{4}}{16\left(1+\beta\right)\left(-2+\beta^{2}\left(\lambda+1\right)\right)^{2}}+$ $\frac{\left(\left(\lambda+1\right)^{2}\beta^{4}+\left(12\lambda^{2}+16\lambda+4\right)\beta^{3}+\left(-12\lambda^{2}-20\lambda-4\right)\beta^{2}+\left(-16\lambda-16\right)\beta+16\lambda+16\right)}{16\left(-2+\beta^{2}\left(\lambda+1\right)\right)^{2}}$ $*\left(a_{0}^{2}+t\sigma^{2}\right)$. Similarly, the manufacturer’s gain from the contract is: $E\left(\pi_{m}^{ASC}\right)=$ $\frac{\left(1-\lambda\right)\left(a_{0}^{2}+t\sigma^{2}\right)\left(\beta-2\right)^{2}}{16-\left(8\lambda+8\right)\beta^{2}}+\frac{\left(1-\eta\right)\left(t\sigma^{2}+a_{0}^{2}\right)\beta^{2}F_{4}}{16\left(1+\beta\right)\left(-2+\beta^{2}\left(\lambda+1\right)\right)^{2}}$.

For the contract to be feasible, it must satisfy the condition $E\left(\pi_{r}^{ASC}\right)>E\left(\pi_{r}^{AN}\right)$.

Setting $\Delta_{10}=E\left(\pi_{r}^{ASC}\right)-E\left(\pi_{r}^{AN}\right)=\frac{\sigma^{2}\left(2-\beta\right)th_{1}}{16\left(-2+\beta^{2}\left(\lambda+1\right)\right)^{2}}+\frac{\eta\left(t\sigma^{2}+a_{0}^{2}\right)\beta^{2}F_{4}}{16\left(\beta+1\right)\left(-2+\beta^{2}\left(\lambda+1\right)\right)^{2}}$. We find the first order derivative of $\Delta_{10}$ with $\eta$, we have $\frac{\partial\Delta_{10}}{\partial\eta}>0$. Solving $\Delta_{10}\left(\eta=\eta_{2}\right)=0$, we find $\eta_{2}=\frac{h_{1}\left(\beta+1\right)\sigma^{2}\left(\beta-2\right)t}{F_{4}\beta^{2}\left(t\sigma^{2}+a_{0}^{2}\right)}$, when $\eta>\eta_{2}$, then $E\left(\pi_{r}^{ASC}\right)>$ $E\left(\pi_{r}^{AN}\right)$. Idem analysis, when i) $\beta<\frac{2}{3}$ and $\lambda\leq\lambda_{2}$; or ii) $\frac{2}{3}\leq\beta<\beta_{3}$ and $0<\lambda<$ $\lambda_{3}$; or iii) $\beta_{3}\leq\beta<1$ and $0<\lambda<\lambda_{1}$, then $h_{1}\leq0$ and $\eta_{2}\geq0$. Besides, find the first order derivative of $\eta_{2}$ with $t$, we have . Solving $\eta_{2}\left(t_{4}\right)=1$, if $t<t_{4}$, then $\eta_{2}<1$, where $t_{4}=\frac{ -a_{0}^{2}\beta^{2}F_{4}}{\sigma^{2}\left(\left(F_{4}-h_{1}\right)\beta^{2}+h_{1}\left(\beta+2\right)\right)}$. Therefore, we cheek whether $\left(F_{4}-h_{1}\right)\beta^{2}+\beta h_{1}+2h_{1}<0$. Setting $h_{4}=\left(F_{4}-h_{1}\right)\beta^{2}+\beta h_{1}+2h_{1}$ $=2\left(2-\beta^{2}\lambda-\beta^{2}\right)\left(3\beta^{3}\lambda\right.+\beta^{3}-5\beta^{2}\lambda+\beta^{2}-4\beta$ $\left.+4\lambda\right)$, if $\lambda<\lambda_{5}$, then $h_{4}<0$, $t_{4}>0$. because of $\frac{t\sigma^{2}}{t\sigma^{2}+a_{0}^{2}}<\frac{1}{2}$, if $\lambda\geq\lambda_{6}$, then $\eta_{2}<\frac{1}{2}$, where $\lambda_{6}=\frac{\beta\left(4-\beta^{2}-\beta\right)}{3\beta^{3}-5\beta^{2}+4}$. Because of $\lambda_{6}<\lambda_{2}$, $\lambda_{6}<\lambda_{3}$, we need to compare $\lambda_{1}$ and $\lambda_{6}$. Setting $\Delta_{\lambda_{16}}=\lambda_{1}-\lambda_{6}$ $=\frac{2\left(2-\beta\right)\left(\beta^{4}+\beta^{3}-8\beta^{2}+4\right)}{3\beta^{5}+\beta^{4}-10\beta^{3}+4\beta^{2}+8\beta}$, analyze it in the same way as above, when $\beta_{5}<\beta<1$, then $\Delta_{\lambda_{16}}<0$; when $\beta_{3}<\beta\leq\beta_{5}$, then $\Delta_{\lambda_{16}}\geq0$.

We conclude that:

(1) In the reselling mode, if $0<t<\min\{1,t_{3}\}$ and $\eta_{1}<\eta<1$, then $E\left(\pi_{r}^{RSC}\right)>$ $E\left(\pi_{r}^{RN}\right)$.

(2) In the agency selling mode, if i) $0<\beta<\frac{2}{3}$, $0<\lambda<\lambda_{6}$, $0<t<\min\left\{1,t_{4}\right\}$ and $\eta_{2}<\eta<1$; or ii) $\frac{2}{3}\leq\beta<\beta_{3}$, $0<\lambda<\lambda_{6}$, $0<t<\min\left\{1,t_{4}\right\}$ and $\eta_{2}<\eta<1$; or iii) $\beta_{3}\leq\beta<1$ , $0<\lambda<\min\left\{\lambda_{1},\lambda_{6}\right\}$, $0<t<\min\left\{1,t_{4}\right\}$ and $\eta_{2}<\eta<1$; Besides, when i) $0<\beta<\frac{2}{3}$, $\lambda_{6}<\lambda\leq\lambda_{2}$ and $\eta_{2}<\eta<1$;or ii) $\frac{2}{3}\leq\beta<\beta_{3}$, $\lambda_{6}<\lambda<\lambda_{3}$ and $\eta_{2}<\eta<1$; or iii) $\beta_{3}\leq\beta<\beta_{5}$, $\lambda_{6}<\lambda<\lambda_{1}$ and $\eta_{2}<\eta<1$, then $E\left(\pi_{r}^{ASC}\right)>E\left(\pi_{r}^{AN}\right)$.

**Proof for Proposition 7:**

The proof is similar to that of Proposition 3.

**Proof for Proposition 8:**

The proof is similar to that of Propositions 1 and 2.

**Proof for Proposition 9:**

The proof is similar to that of Proposition 3.
